# Non-Systemic Oral Cellulose-Based Superabsorbent Hydrogels for Weight Management: Potential Drug-Device Interactions Estimated by a Simplified In Vitro Assay

**DOI:** 10.3390/pharmaceutics18091173

**Published:** 2026-09-17

**Authors:** Eliana Panteca, Ivo Surano, Christian Demitri, Yishai Zohar, Marta Madaghiele, Alessandro Sannino

**Affiliations:** 1 Gelesis, 73021 Calimera, Italy; isurano@gelesis.com (I.S.);; 2 Department of Experimental Medicine, University of Salento, 73100 Lecce, Italy; christian.demitri@unisalento.it (C.D.); alessandro.sannino@unisalento.it (A.S.)

**Keywords:** superabsorbent hydrogel, obesity, weight management, drug–device interactions, UV-Vis spectroscopy

## Abstract

**Background/Objectives:** A technological platform of non-systemic oral cellulose-based superabsorbent hydrogels (CB-SAHs) has been recently developed as novel therapeutics for treating obesity. Further clinical advancement of CB-SAH candidates from this platform requires evaluating their potential impact on the absorption of co-administered drugs. **Methods:** In this work, we developed a simplified in vitro assay to assess whether and to what extent given CB-SAHs may interact with co-administered drugs. We selected two different CB-SAHs and tested them in contact with various solubilized drug substances under simulated gastrointestinal conditions. A total of 23 drugs were chosen among those that could be used to treat frequent conditions associated with excess weight (e.g., type 2 diabetes) or to deal with common therapeutic needs (e.g., pain relief). Final drug recovery (FDR) in the simulated intestinal medium was quantified using UV-Vis spectroscopy to assess drug–hydrogel interactions. **Results:** While most drugs achieved an average FDR exceeding 80% with both hydrogels, six compounds exhibited lower FDR values (~68–78%) with at least one CB-SAH, indicating stronger, specific drug–hydrogel interactions. Notably, the FDR measured for metformin with both CB-SAHs (~94%) was consistent with previous clinical findings demonstrating negligible metformin–CB-SAH interaction. **Conclusions:** Although simplified, the in vitro assay shows promise as an exploratory screening tool to identify specific drug–CB-SAH interactions that warrant subsequent clinical evaluation.

## 1. Introduction

The assessment of drug–device interactions (DDIs) is extremely important in the development of medical devices for in vivo use. Some drugs, for instance, may alter the functionality of given devices (e.g., implantable glucose monitoring systems [1], pacemakers [2] and implantable cardioverter-defibrillators [3]), thus the evaluation of potential undesired interplays is a prerequisite for risk assessment and safety. Similarly, the performance of some devices (e.g., inhalers [4]) may affect the bioavailability of selected drugs, i.e., the rate and concentration at which the drugs enter the systemic circulation. The study of the effects of drugs on devices and/or of devices on drugs should include both in vitro and in vivo tests. Although in vitro tests cannot recapitulate and replace in vivo ones, they are very helpful to identify potential DDI risks, thus providing useful indications for the design of in vivo studies [1].

Recently, non-systemic oral superabsorbent hydrogels (SAHs) have gained increasing interest as potential diagnostic and/or therapeutic tools that target the gastrointestinal (GI) tract [5,6,7,8,9,10,11,12]. Due to their unique superabsorbent capacity, SAHs enable the realization of GI devices that can be orally delivered in a practical number of capsules and then significantly expand by means of hydration when arriving to the stomach or the gut. SAH-based devices are designed to show given volume, shape, and mechanical elasticity (i.e., firmness) within the GI tract for predetermined periods of time, which can be properly adjusted depending on the intended use [5,6,7,8,9,10,11,12].

**Figure 1:**
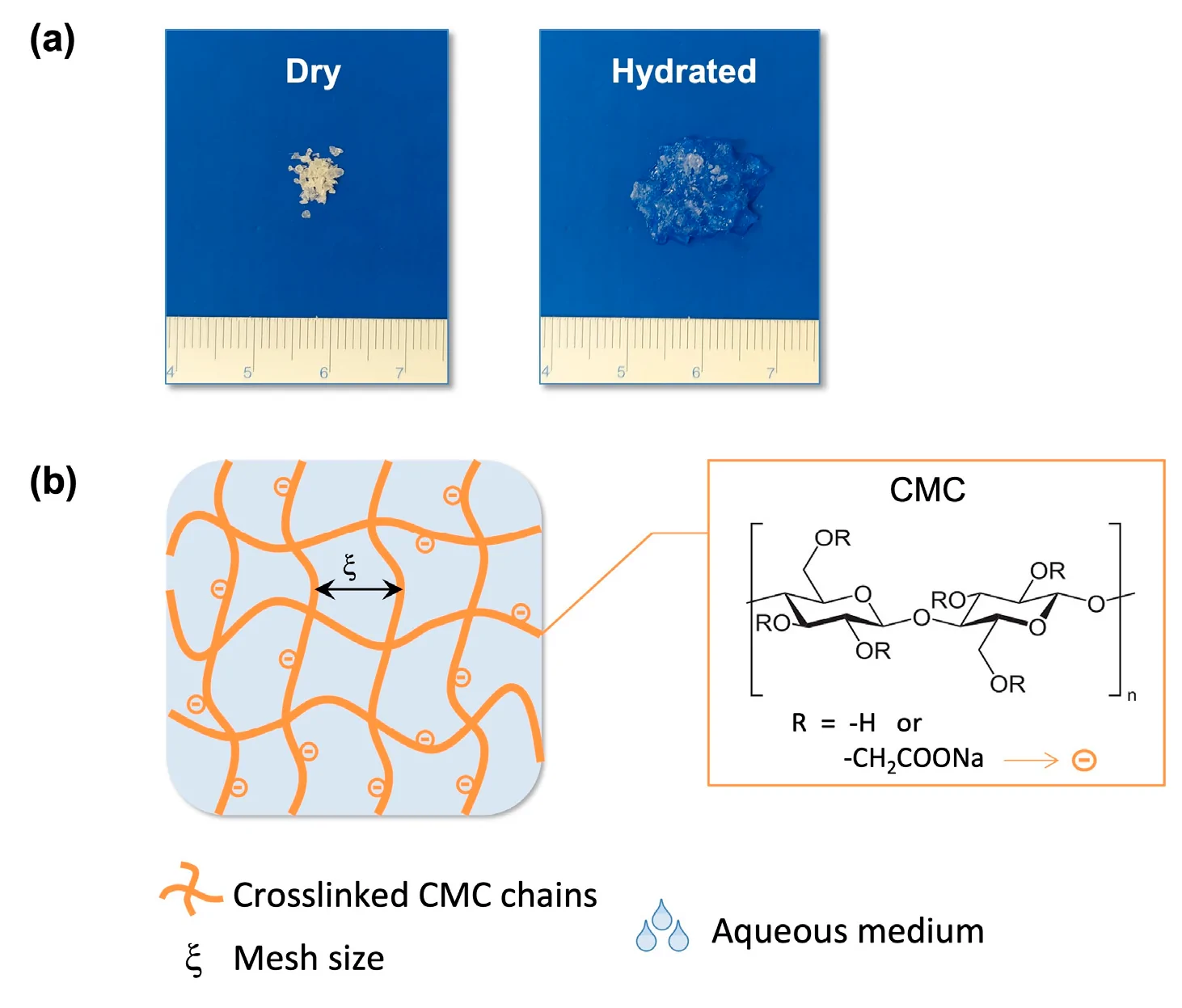
Structure of CB-SAHs. (**a**) Physical appearance of a few CB-SAH granules, with a size range of 100–1000 µm, before and after hydration. (**b**) Scheme of the CB-SAH microstructure upon hydration, with crosslinked chains of carboxymethylcellulose sodium salt (CMC) forming a polyanionic network of given mesh size.

In this context, inspired by the composition and mechanical properties of ingested raw vegetables, we previously developed a technological platform of non-systemic oral cellulose-based superabsorbent hydrogels (CB-SAHs) as a new therapeutic approach for treating obesity [9,13,14,15]. The products of this technology (Figure 1) are the first reported SAHs to be exclusively based on naturally derived, generally recognized as safe (GRAS) components used in foods, i.e., carboxymethylcellulose sodium salt (CMC) chemically crosslinked with citric acid [9]. By using an in vitro GI model, we showed that the CB-SAHs, taken before meals, hydrate in the stomach (up to about 100 times their original volume) to form thousands of small gel pieces without caloric value, which emulate basic compositional and mechanical features of ingested raw vegetables [9]. Like raw vegetables, the gel pieces reduce the caloric density of the meal and increase the volume and the elasticity of the ingested foods throughout the stomach and the small intestine, thus working to enhance satiety and assist in weight management, mainly through mechanobiological modes of action. When arriving to the colon, the gel pieces finally undergo a partial degradation promoted by bacterial enzymes. This allows the carried water to be returned to the body, which is important for safety, while the cellulosic material is expelled in the feces [9]. Since the CB-SAHs remain undigested in the stomach and the small intestine, the study of potential interactions with co-administered drugs (especially drugs taken with meals or close to meals) is essential for risk assessment and further clinical development. The gel pieces may indeed modify the rate and/or the degree of drug absorption in the GI tract.

In this work, we developed a simplified in vitro screening assay to detect potential interactions between candidate CB-SAHs and various oral drugs, thus establishing a framework for further DDI evaluations. Specifically designed to replicate the concurrent GI transit of CB-SAHs and co-administered drugs, the assay relied on initial drug solubilization and absorption by the hydrogel under simulated gastric conditions to facilitate the physical interaction. The amount of free drug recovered in the simulated intestinal environment was then quantified using ultraviolet–visible (UV-Vis) spectroscopy to identify specific drug–hydrogel interactions.

## 2. Materials and Methods

### 2.1. Chemicals

All chemicals used to prepare simulated gastric fluid (SGF), simulated intestinal fluid (SIF) and dissolution media were of analytical grade and purchased from Sigma Aldrich (Milano, Italy). Purified water was produced using a Milli-Q purification system (Merck Millipore, Burlington, MA, USA). Drug substances selected for the drug–hydrogel interaction study are detailed in Table 1 [16].

### 2.2. Simulated Gastric and Intestinal Fluids

To simulate upper GI tract environments, enzyme-free stock solutions of SGF (pH 1.1) and SIF (pH 6.8) were prepared according to United States Pharmacopeia (USP) specifications (Supplementary Table S1). Reflecting CB-SAH capsule administration with two cups of water, a stock solution of diluted SGF (SGF/water 1/8 *v*/*v*, pH 2.1) was also prepared to more accurately mimic the gastric environment during CB-SAH hydration.

### 2.3. Cellulose-Based Superabsorbent Hydrogels (CB-SAHs)

Two granular CB-SAHs with particle sizes ranging from 100 to 1000 μm, referred to as GelA and GelB, were synthesized using a proprietary technological platform, as previously described [9]. As summarized in Table 2, both CB-SAHs consist of carboxymethylcellulose sodium salt (CMC) crosslinked with citric acid but differ in the type of CMC used (with reference to the molecular weight distribution) and the post-hydration elasticity in diluted SGF. For each CB-SAH, 12 samples (3.5 g each) from 3 independent batches were used to evaluate the drug–hydrogel interactions, according to the protocol outlined below.

### 2.4. In Vitro Drug–Hydrogel Interaction Assay

**Figure 2:**
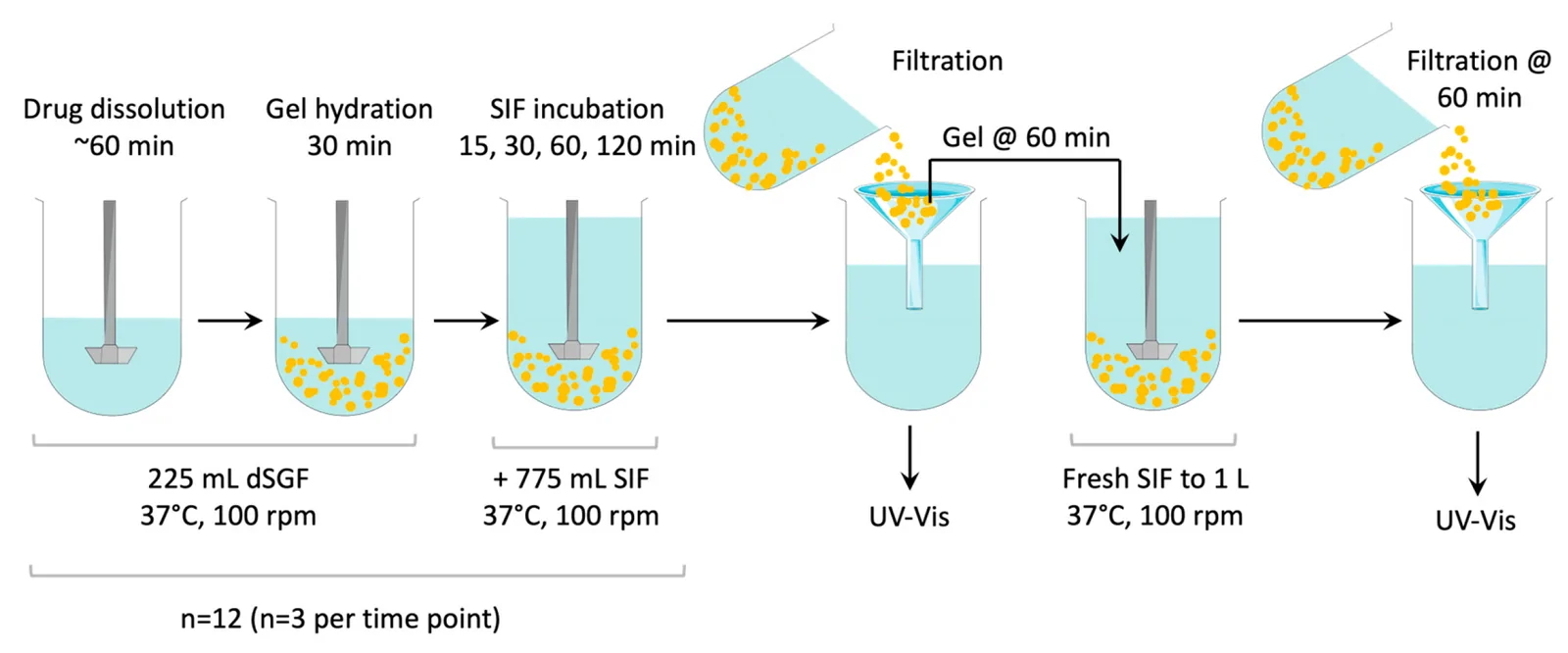
Schematic illustration of the in vitro drug–hydrogel interaction assay. Step 1: The drug is dissolved in 225 mL of diluted SGF (dSGF). Step 2: CB-SAH is added to each vessel (n = 12) to absorb the entire medium and dissolved drug. Step 3: After 30 min, 775 mL of SIF is added to emulate gastric emptying; following sample filtration at 15, 30, 60, or 120 min (n = 3 at each time point), free drug in solution is measured via UV-Vis analysis. Step 4: The gel samples collected at 60 min (Step 3) are further incubated in fresh SIF for an additional 60 min to emulate intestinal fluid secretion, after which the released drug is measured to calculate total recovery. All steps are performed at 37 °C under mechanical stirring (100 rpm). For clarity, drug-free and gel-free controls are omitted from the diagram.

To emulate the sequential phases of digestion, we designed a simplified, multi-step in vitro assay, in which a hydrogel and a target drug interact under simulated gastric conditions before transitioning to a simulated small intestinal environment for the expected in vivo residence time [9]. As illustrated in Figure 2, the drug is initially dissolved in 225 mL of diluted SGF (pH = 2.1), under continuous stirring at 37 °C (Step 1, n = 12). A CB-SAH sample (3.5 g) is then added to each drug solution and incubated for 30 min (Step 2). Given the hydration capacity of the CB-SAH in diluted SGF (approximately 85 and 77 g/g for GelA and GelB respectively; Table 2), this 225 mL volume ensures total absorption of the gastric medium and dissolved drug, while leaving minimal residual hydration capacity. Step 2 thus establishes an interaction-promoting environment that facilitates physical binding of the absorbed drug to the polymer matrix.

To simulate gastric emptying, 775 mL of SIF is then added (Step 3). This volume provides a large excess of intestinal medium, reproducing the gastric-to-intestinal transition within the constraints of the simplified in vitro setup. The resulting total volume of 1 L, corresponding to the maximum vessel capacity, yields a final pH of 6.8 ± 0.1, representative of the intestinal milieu. Samples are then incubated in this simulated intestinal environment for 15, 30, 60, or 120 min (n = 3 per time point). The amount of free drug in the intestinal medium is quantified at each time point via UV-Vis spectroscopy to evaluate drug–hydrogel interactions over time.

Finally, to emulate continuous intestinal fluid secretion (Step 4), the CB-SAH samples collected at 60 min in Step 3 are incubated with fresh SIF for an additional hour, after which the released drug is measured to estimate the final recovery.

The assay setup also includes two experimental controls that undergo the same gastric-to-intestinal transition: a drug-only, CB-SAH-free reference to account for drug dissolution and quantification, and a drug-free, CB-SAH-only control to evaluate potential UV-Vis absorbance from any uncrosslinked CMC released by the hydrogel matrix.

#### 2.4.1. Drug Dissolution and Quantitation via UV-Vis Spectroscopy

To perform the in vitro assay, suitable conditions for drug solubilization and UV-Vis analysis in simulated gastric and intestinal media were first established [17,18,19,20].

The specified dose (Table 1) was added to 225 mL of diluted SGF (dSGF) at 37 °C and stirred using the USP Apparatus 2 (paddle method, 100 rpm) until a clear solution was obtained. For poorly soluble drugs, primarily belonging to Biopharmaceutical Classification System (BCS) Classes II and IV [16], the medium was slightly modified by adding a proper amount of surfactant (in the range 0.1–2% *w*/*w*), in accordance with the Food and Drug Administration (FDA) guidelines [17]. If the surfactant alone failed to achieve complete dissolution within 120 min of stirring, the drug was pre-treated with 5 mL of an organic solvent to facilitate solubilization in dSGF (Supplementary Table S1). Upon complete dissolution, a 5 mL aliquot was analyzed by UV-Vis spectroscopy in the wavelength range 200–800 nm, using drug-free dSGF (or modified dSGF) as a blank. All UV-Vis spectra were acquired by means of a Cary 60 spectrophotometer (Agilent, Santa Clara, CA, USA). The wavelength of maximum absorbance was selected as a reference to quantify the drug concentration. If necessary, samples were appropriately diluted with medium to obtain a maximum absorbance of about 1.0 AU. Drug stability in the medium was also verified by monitoring the UV-Vis absorbance for up to 24 h to determine the valid analytical time window.

Following drug dissolution under simulated gastric conditions, drug solubility, detection, and stability were evaluated in the simulated intestinal environment. Briefly, the drug was dissolved in 225 mL of dSGF at 37 °C, followed by the addition of 775 mL of SIF to simulate gastric emptying. For sparingly soluble drugs, SIF was supplemented with the same surfactant concentration used in dSGF. After stirring for at least 1 h, a 5 mL aliquot was withdrawn and scanned across 200–800 nm to determine the maximum absorbance peak, using drug-free medium (or modified medium) as a blank; if necessary, samples were diluted with medium to achieve an absorbance of approximately 1.0 AU. Solution stability was confirmed by monitoring UV-Vis absorbance for up to 24 h.

Solutions with known drug concentrations, obtained via serial dilutions, were finally used to determine a linear calibration curve correlating the absorbance to the drug concentration (Supplementary Figure S1).

#### 2.4.2. Step 1—Drug Dissolution in Simulated Gastric Environment

Having established the experimental conditions for drug dissolution and UV-Vis detection, the in vitro drug–hydrogel interaction assay was conducted. The selected drug dose was first dissolved in 225 mL of dSGF at 37 °C. Twelve replicate solutions were prepared in independent vessels to evaluate the drug–hydrogel interactions (Figure 2), together with an additional vessel as a drug-only control. UV-Vis measurements were then performed to determine the absorbance at the wavelength of interest. The mean absorbance across these samples served as a reference to calculate the starting drug concentration in each vessel:

(1) $${Drug\;in\;solution}_{STEP1}=Dosage\;\times \;\frac{{Sample\;Absorbance}_{STEP1}}{{Mean\;Absorbance}_{STEP1}}$$

#### 2.4.3. Step 2—CB-SAH Hydration in Simulated Gastric Environment

After drug dissolution, 3.5 g of dry CB-SAH (GelA or GelB) was added to each vessel (excluding the drug-only control) and allowed to hydrate for 30 min under continuous stirring at 37 °C to completely absorb the liquid medium. A CB-SAH control sample was separately incubated in drug-free dSGF.

#### 2.4.4. Step 3—Drug Recovery upon Simulated Gastric Emptying

Then, 775 mL of SIF or modified SIF at 37 °C was added to all vessels under continuous stirring. At each designated fixed time point (15, 30, 60 or 120 min) three CB-SAH samples were separated from the liquid medium via filtration, and testing for those specific vessels was terminated. The volume of liquid medium recovered from each vessel was recorded to evaluate potential volume changes resulting from residual CB-SAH absorption in the intestinal milieu. A 5 mL aliquot was then analyzed by UV-Vis spectroscopy, and the free drug concentration was calculated using the corresponding calibration curve (Supplementary Figure S1).

To account for potential UV-Vis interference from released CMC, the drug-free, CB-SAH-only control was analyzed at each time point. When appreciable CMC absorbance was observed at the target wavelength; this background value was subtracted from the experimental sample absorbance prior to quantifying the drug in solution. The percentage of drug recovery (DR) at the designated time point, with respect to the initial amount of dissolved drug, was then calculated as follows:

(2) $${DR}_{\left(t\right)}=\frac{{Drug\;in\;solution}_{STEP3(t)}}{{Drug\;in\;solution}_{STEP1}}\times 100$$

Variations of DR over time were considered indicative of the drug diffusion kinetics through the CB-SAH matrix, which depended on specific drug–hydrogel interactions.

#### 2.4.5. Step 4—Drug Recovery upon Simulated Secretion of Intestinal Fluids

To simulate additional intestinal fluid secretion, the CB-SAH samples collected at 60 min in Step 3 (n = 3) were added with fresh SIF (or modified SIF) at 37 °C to reach a final volume of 1 L and incubated for additional 60 min (Figure 2). As described for Step 3, the hydrogel and liquid medium were separated via filtration, and the free drug in solution was quantified by UV-Vis spectroscopy. Background CMC interference was accounted for where applicable. For select drug–hydrogel combinations, a distinct calibration curve was applied to maintain linearity at lower concentration ranges (Supplementary Figure S1).

For each sample the cumulative or final drug recovery (FDR) in the simulated intestinal environment was calculated as

(3) $$FDR=\frac{{Drug\;in\;solution}_{STEP3\;(60)}+{Drug\;in\;solution}_{STEP4}}{{Drug\;in\;solution}_{STEP1}}\times 100$$

and used to evaluate overall drug–hydrogel interactions under the tested conditions.

### 2.5. Statistical Analysis

Results were expressed as mean ± standard deviation (SD). Statistical analyses were performed using Jamovi software (version 2.7.38). Two-way analysis of variance (ANOVA) was conducted to evaluate and compare the final drug recovery values between the two different CB-SAHs across the 23 distinct drugs. Drug type and gel type were used as fixed factors, investigating their main effects and interaction. Bonferroni adjusted post hoc tests were then performed to compare GelA versus GelB within each specific drug context. For all analyses, statistical significance was defined as *p* < 0.05.

## 3. Results

### 3.1. Drug Dissolution

As detailed in Section 2 and shown in Figure 2, the in vitro assay required complete preliminary dissolution of the drug in diluted SGF. Supplementary Table S2 summarizes the experimental parameters used to dissolve each drug substance. Briefly, drugs belonging to BCS Classes I and III (Table 1) dissolved in the simulated gastric medium at 37 °C within 30–60 min of stirring, with the exception of levothyroxine, which required 120 min and the addition of 1% *w*/*w* sodium lauryl sulfate (SLS). Similar surfactant concentrations and dissolution times were also necessary for most remaining drugs, whereas bendroflumethiazide, hydrochlorothiazide, and gemfibrozil needed a pre-incubation in 5 mL of methanol prior to incubation in diluted SGF. The resulting drug solutions were visually inspected to confirm optical clarity and the absence of particulates; drug stability was also verified by monitoring the UV-Vis absorbance for up to 24 h. Furthermore, the addition of SIF to emulate the gastric-to-intestinal transition (Step 3, Figure 2) induced no drug precipitation or degradation, as confirmed by absorbance measurements of the drug-only control. Therefore, any reduced drug recovery observed in the presence of CB-SAHs in simulated intestinal conditions was attributable to specific drug–hydrogel interactions rather than incomplete drug dissolution or precipitation.

### 3.2. Drug Recovery upon Simulated Gastric Emptying (Step 3)

Time-dependent DR values in the simulated intestinal environment (provided for each drug–hydrogel combination in Supplementary Figure S2) remained relatively constant for most drugs, generally exceeding 60% at all time points (15, 30, 60 and 120 min) with minimal differences between GelA and GelB. Notably, average DR values for lovastatin, rosuvastatin, rivaroxaban, and repaglinide reached or exceeded 90% at all time points with both CB-SAHs. In contrast, DR values for carvedilol, hydrochlorothiazide and acarbose remained below 60% throughout the 2 h incubation with both hydrogels. Carvedilol and hydrochlorothiazide exhibited stable DR values between 50% and 60% at each time point, whereas acarbose displayed a progressive DR increase (~48–60% with GelA and ~35–60% with GelB). These findings suggested a reduced or delayed drug diffusion through the CB-SAH network.

### 3.3. Drug Recovery upon Simulated Secretion of Intestinal Fluid (Step 4)

**Figure 3:**
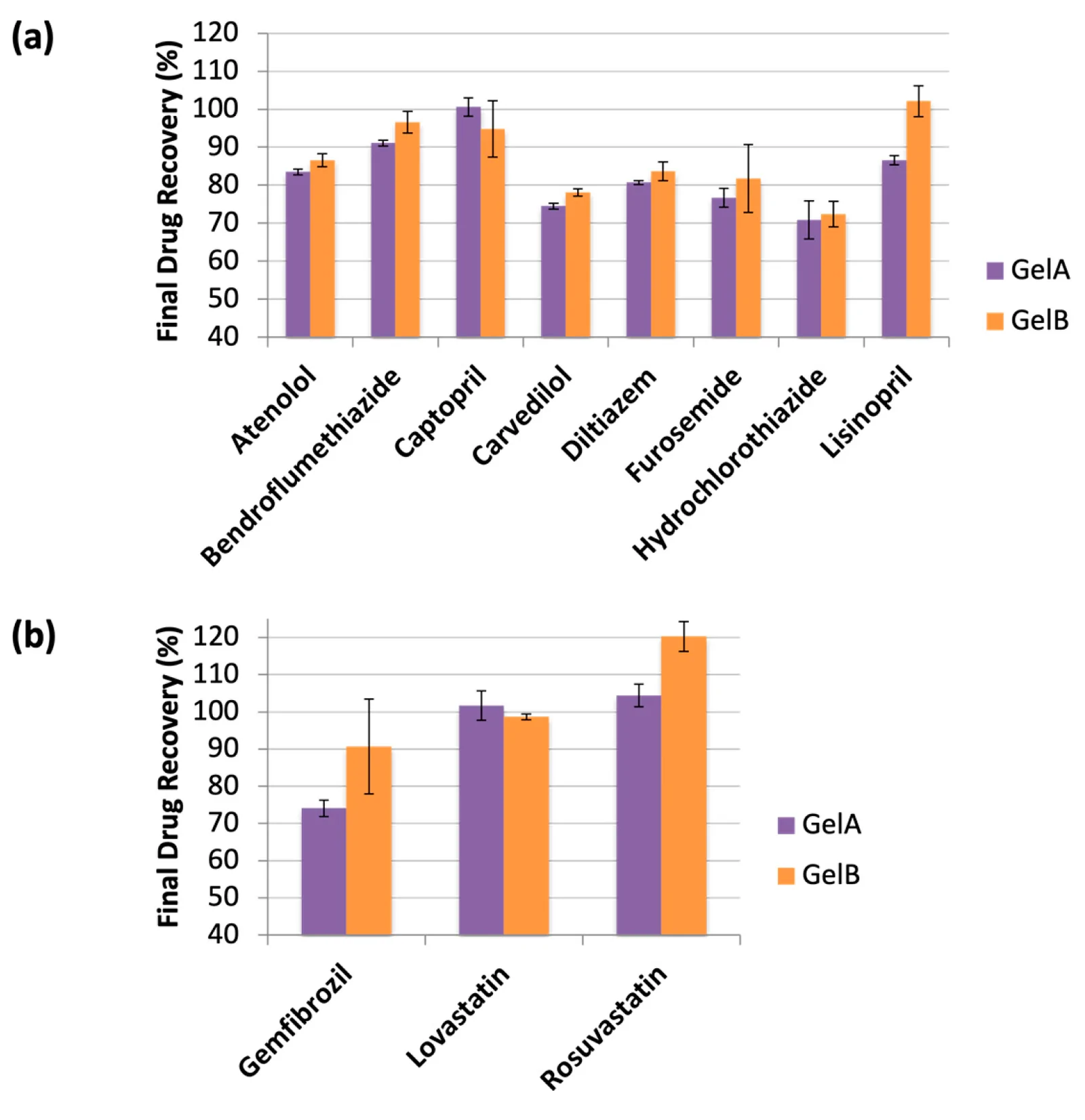

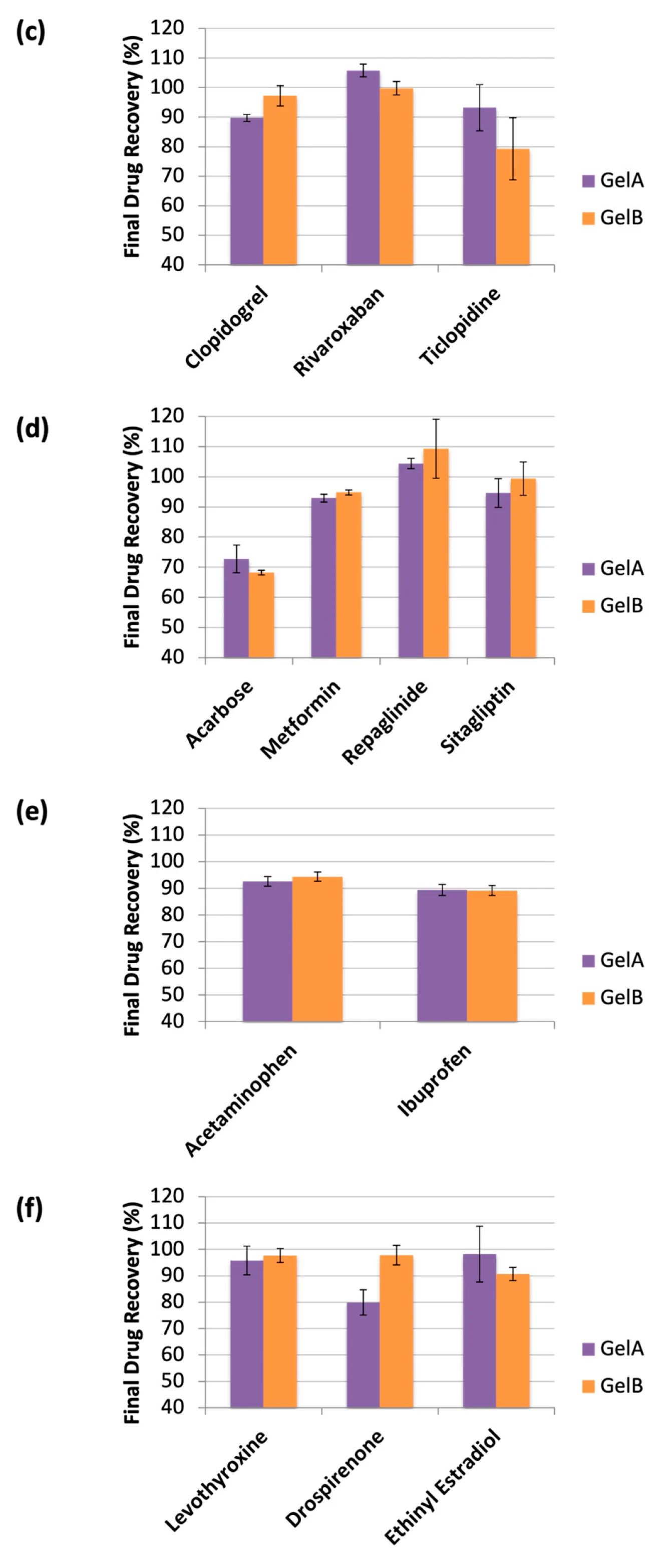
Bar plots of final drug recovery (FDR) in simulated intestinal environment (mean ± SD; n = 3). For clarity, plots are grouped by the drug therapeutic indication: (**a**) hypertension; (**b**) dislypidemia; (**c**) blood clotting; (**d**) type 2 diabetes; (**e**) pain; (**f**) hypothyroidism (levothyroxine) and contraception (drospirenone and ethinyl estradiol).

For each drug–hydrogel combination, the final drug recovery (FDR) achieved in the simulated intestinal medium at Step 4 (Figure 2) was quantified to assess the extent of drug–hydrogel interaction. The experimental results are summarized in Table 3 and Figure 3, with the latter presenting FDR bar plots categorized by therapeutic indication.

For most drugs, an average FDR exceeding 80% was achieved with both GelA and GelB, with comparable values observed between the two CB-SAHs. Notably, certain FDR values exceeded 100%. Minor elevations up to 102%, such as those observed for captopril, lisinopril, and lovastatin, fell within the analytical accuracy of the UV-Vis methods and were interpreted as complete recovery, consistent with the high DR values observed at Step 3 (Supplementary Figure S2). In contrast, larger FDR values, such as those obtained for rosuvastatin, rivaroxaban, and repaglinide (Table 3), indicated drug-specific analytical limitations, likely arising from the commercial medicinal products used in the in vitro assay (Table 1). As rosuvastatin, rivaroxaban, and repaglinide belong to BCS Class II, their commercial formulations may contain excipients that could absorb light at the detection wavelength, falsely increasing the measured absorbance and yielding FDR values above 100%. While the UV-Vis methods accounted for potential CMC interference and medium composition variations, background absorbance from specific formulation excipients could not be isolated.

Among the tested drugs, only six (carvedilol, furosemide, hydrochlorothiazide, gemfibrozil, ticlopidine, and acarbose) exhibited FDR values within the approximate range of 68–78% with at least one CB-SAH (Table 3). In particular, the lowest FDR values were recorded for carvedilol, hydrochlorothiazide, and acarbose with both GelA and GelB, consistent with the DR findings.

A two-way ANOVA was conducted to analyse the effects of drug type and CB-SAH type on the FDR. The analysis showed that both main factors significantly influenced the FDR (*p* < 0.001 for both drug and CB-SAH type). Furthermore, a significant interaction between the two factors was observed (*p* < 0.001). Bonferroni-adjusted post hoc tests comparing GelA and GelB for each drug confirmed that most drugs exhibited comparable FDR values between the two hydrogels (*p* > 0.05), with the exceptions of gemfibrozil and drospirenone. These two drugs showed significantly higher FDRs with GelB than with GelA, respectively 90.7 ± 12.7% vs. 74.1 ± 2.2% for gemfibrozil (*p* = 0.044), and 97.8 ± 3.7% vs. 80.0 ± 4.8% for drospirenone (*p* = 0.012).

## 4. Discussion

In this study, we present an in vitro drug–hydrogel interaction assay specifically designed to simulate the GI transit of non-systemic oral CB-SAHs with co-administered drugs (Figure 2). While in vitro drug–hydrogel interactions are typically investigated to evaluate hydrogel performance in controlled drug delivery systems [21,22,23], the primary objective of this work was to establish a simplified screening method to detect potential drug–CB-SAH interactions and guide subsequent clinical evaluations.

We hypothesized that these interactions depend on the specific CMC used as the CB-SAH matrix (Figure 1 and Table 2), as well as the nature of the co-administered drug. To test this hypothesis, the assay evaluated multiple drug–hydrogel combinations comprising two distinct CB-SAH formulations (GelA and GelB; Table 2) and 23 drug substances (Table 1). As illustrated in Figure 2 and detailed in Section 2, the fundamental premise of the study required complete drug dissolution and interaction with the CB-SAH prior to reaching the simulated intestinal environment. Final drug recovery (FDR) in the simulated intestinal medium served as a quantitative parameter to identify specific drug–CB-SAH interactions that may warrant further clinical investigation.

Assay results (Table 3 and Figure 3) demonstrated that FDR was significantly influenced by both drug type and CB-SAH type. The high FDR values (>80%) observed for most drugs indicated limited interaction with both CB-SAHs under the tested conditions. However, a subset of six drugs exhibited lower FDR values (68–78%) with at least one CB-SAH, thus suggesting stronger and specific drug–hydrogel interactions. These included the antihypertensives carvedilol, furosemide, and hydrochlorothiazide, the lipid-regulating agent gemfibrozil, the antithrombotic ticlopidine, and the antidiabetic acarbose. Interestingly, significant FDR differences between GelA and GelB for a given drug were detected only for drospirenone and gemfibrozil (Table 3). Overall, these findings suggest that the specific drug–CB-SAH interactions observed in this assay are primarily driven by the common CMC polymer backbone and, to a lesser extent, by the specific CMC type utilized.

Although this in vitro assay cannot directly elucidate the underlying mechanism of drug–hydrogel interactions, we hypothesized that various physical interactions occur following drug dissolution and uptake by the CB-SAH. For instance, negative electrostatic charges on the CMC backbone (Figure 1b) may attract positively charged groups on co-administered drug molecules, retaining them within the polymer network. Indeed, drug release from hydrogel matrices is well documented to be significantly reduced or delayed when the drug and polymer bear complementary charges [21,22], whereas repulsive forces between like charges seem to exert a minimal influence on drug release [21,22]. Electrostatic or ionic interactions occurring during the simulated GI transit are strongly affected by the ionization states of both the hydrogel and the drug, which in turn depend on local pH and ionic strength. Additional physical drug–hydrogel interactions during the assay may result from hydrophobic associations, where unsubstituted domains along the CMC backbone serve as potential binding sites for lipophilic drugs [23]. Furthermore, drug retention and release kinetics may be modulated by the CMC network morphology, such as degree of crystallinity and mesh size (which depends on matrix hydration and elasticity), thereby restricting molecular mobility through the CB-SAH matrix (Figure 1b) [23]. Consequently, further in vitro analyses are necessary to shed light on the specific interaction mechanisms observed for these drug–hydrogel combinations (Table 3), as well as the contribution of uncrosslinked CMC to drug diffusion.

Although capable of detecting specific drug–hydrogel interactions, the in vitro assay presents several limitations. First, simulating the GI transit of CB-SAHs and co-administered drugs represents an oversimplification of the complex in vivo gastrointestinal environment, yielding results that do not necessarily predict clinical drug–device interactions (DDIs) or reduced systemic drug availability. Second, the assay assumes complete drug dissolution in the gastric environment prior to hydrogel uptake. This assumption, while intended to maximize the in vitro interaction potential, poorly mirrors the in vivo physiology, particularly for low-solubility drugs (BCS Classes II and IV). Therefore, significant in vitro drug–hydrogel interactions detected here, such as those observed for carvedilol, furosemide, hydrochlorothiazide, gemfibrozil, ticlopidine, and acarbose, may not occur in vivo or translate to clinically relevant interactions. Third, utilizing UV-Vis spectroscopy to quantify the drug recovery may lack sufficient accuracy and selectivity for certain compounds, particularly when commercial pharmaceutical formulations are evaluated instead of pure reference standards (Table 1). In such cases, excipient absorbance interference can falsely increase FDR values above 100%, as observed for rosuvastatin, rivaroxaban, and repaglinide. Additionally, compounds administered at low clinical doses may yield insufficient UV-Vis signal. Consequently, doses of repaglinide, levothyroxine, drospirenone, and ethinyl estradiol were increased compared to standard clinical doses to facilitate the detection (Table 1). While UV-Vis spectroscopy was selected for its simplicity and cost-effectiveness compared to techniques such as high-performance liquid chromatography (HPLC) [18], incorporating HPLC into this in vitro assay protocol is recommended to enhance analytical sensitivity and specificity.

Despite the limitations discussed above, this study allowed identifying six drug candidates for further clinical evaluation: carvedilol, furosemide, hydrochlorothiazide, gemfibrozil, ticlopidine, and acarbose. Because interactions observed in vitro may not necessarily occur or have clinical relevance in vivo (particularly for drugs taken with food), these findings warrant targeted clinical studies to evaluate the impact of CB-SAH candidates on the systemic absorption of these drugs. Notably, for metformin, which yielded FDR values of ~94% with both GelA and GelB (Table 3), a previous clinical study demonstrated that a CB-SAH product derived from the same technological platform had no significant effect on metformin pharmacokinetics (PK) beyond that of food [14]. In that study, a comparable in vitro assay, utilizing HPLC as the analytical method and minor differences in the experimental setup, similarly yielded metformin recoveries between 83% and 94% in simulated intestinal conditions. Thus, the high FDR values observed in this work, which indicated negligible metformin–CB-SAH interaction, correlate well with prior HPLC data and in vivo clinical findings. For other drugs, further in vitro and in vivo studies remain necessary to validate these assay findings and evaluate their potential translational implications.

## 5. Conclusions

In this study, we developed a simplified in vitro assay to screen for potential interactions between novel non-systemic oral CB-SAH candidates for weight management (GelA and GelB) and co-administered drugs. Across 23 drug substances, both hydrogels exhibited moderate interactions with six compounds (carvedilol, furosemide, hydrochlorothiazide, gemfibrozil, ticlopidine, and acarbose), whereas minor or negligible interactions were observed for the remaining drugs. Although replacing UV-Vis spectroscopy with higher-resolution analytical methods such as HPLC would enhance quantitative accuracy and selectivity, this assay offers a rapid and accessible screening platform to identify specific drug–CB-SAH combinations for subsequent clinical investigation.

## Figures and Tables

**Table 1:** Drug substances and doses utilized for the drug–hydrogel interaction study. The use of a pharmaceutical reference standard (Std) or a medicinal product (Med) is specified; where both are indicated, the Std was used to set up the UV-Vis quantification method, and the Med was used to perform the interaction study. Doses correspond to standard oral formulations except in the cases of repaglinide, levothyroxine, drospirenone, and ethinyl estradiol, where the minimum amount required for UV-Vis detection was used.

Therapeutic Indication	Drug Substance	DrugBank ID	BCS Class *	Std/Med	Manufacturer or Supplier	Dose (mg)
Hypertension	Atenolol	DB00335	III	Std	Sigma Aldrich	50
	Bendroflumethiazide	DB00436	n.a.	Std	Sigma Aldrich	10
	Captopril	DB01197	III	Std	Fluka Analytical (Buchs, Switzerland)	25
	Carvedilol	DB01136	II	Med	Doc Generici (Milano, Italy)	25
	Diltiazem	DB00343	I	Std	Sigma Aldrich	60
	Furosemide	DB00695	IV	Std	Fluka Analytical	40
	Hydrochlorothiazide	DB00999	IV	Std	Sigma Aldrich	100
	Lisinopril	DB00722	III	Med	Sandoz (Basel, Switzerland)	40
Dyslipidemia	Gemfibrozil	DB01241	II	Med	EG EuroGenerici (Milano, Italy)	600
	Lovastatin	DB00227	II	Std	Fluka Analytical	40
	Rosuvastatin	DB01098	II	Med	AstraZeneca (Cambridge, UK)	40
Blood clotting	Clopidogrel	DB00758	II	Med	Sanofi Aventis (Paris, France)	75
	Rivaroxaban	DB06228	II	Med	Bayer (Leverkusen, Germany)	20
	Ticlopidine	DB00208	n.a.	Std	Sigma Aldrich	250
Type 2 diabetes	Acarbose	DB00284	III	Std/Med	Fluka Analytical/Bayer	100
	Metformin	DB00331	III	Med	Doc Generici	1000
	Repaglinide	DB00912	II	Med	Mylan Generici (Canonsburg, PA, USA)	30
	Sitagliptin	DB01261	III	Med	MSD (Rahway, NJ, USA)	100
Pain	Acetaminophen	DB00316	I	Std	Sigma Aldrich	650
	Ibuprofen	DB01050	II	Med	Angelini (Rome, Italy)	200
Hypothyroidism	Levothyroxine	DB00451	III	Med	Merck Serono (Darmstadt, Germany)	2
Contraception	Drospirenone **	DB01395	n.a.	Std/Med	EDQM/Bayer (Strasbourg, France)	6
	Ethinyl Estradiol **	DB00977	n.a.	Std/Med	Sigma Aldrich/Bayer	20

* BCS Class refers to the Biopharmaceutical Classification System, where drugs are divided into four classes based on their solubility (high for Classes I and III, low for Classes II and IV) and intestinal permeability (high for Classes I and II, low for Classes III and IV). ** Drospirenone and ethinyl estradiol were analyzed simultaneously, as they are combined in a single contraceptive product. n.a. = not available.

**Table 2:** Composition, absorption capacity and elasticity of GelA and GelB. Two CMC types with fixed degree of substitution, i.e., the average number of carboxymethyl groups per glucose unit (0.7), and different average molecular weight (MW) and polydispersity index (PDI), were used.

CB-SAH	CMC Type (MW/PDI)	Citric Acid/CMC (% Weight)	MUR * (g/g)	Elasticity G′ * (kPa)
GelA	CMC-L (2.0 × 10^6^ Da/11)	0.3	85.3 ± 1.1	1.06 ± 0.09
GelB	CMC-H (2.5 × 10^6^ Da/4.2)	0.2	77.0 ± 1.0	1.85 ± 0.14

* Values of medium uptake ratio (MUR, i.e., absorbed grams of liquid medium per gram of dry material) and elasticity (i.e., the elastic modulus G′) refer to the CB-SAHs’ hydration in diluted SGF (mean ± SD; n = 3).

**Table 3:** In vitro assay results: final drug recovery (FDR) values yielded in simulated intestinal environment for the tested drug–hydrogel combinations (mean ± SD; n = 3).

Therapeutic Indication	Drug	FDR %	
		GelA	GelB
Hypertension	Atenolol	83.5 ± 0.8	86.6 ± 1.7
	Bendroflumethiazide	91.1 ± 0.8	96.6 ± 2.8
	Captopril	100.6 ± 2.4	86.6 ± 1.7
	Carvedilol	74.5 ± 0.8	78.1 ± 0.9
	Diltiazem	80.7 ± 0.5	83.7 ± 2.5
	Furosemide	76.7 ± 2.5	81.8 ± 8.9
	Hydrochlorothiazide	70.9 ± 5.0	72.4 ± 3.3
	Lisinopril	86.6 ± 1.2	102.1 ± 4.0
Dyslipidemia	Gemfibrozil *	74.1 ± 2.2	90.7 ± 12.7
	Lovastatin	101.7 ± 4.0	98.7 ± 0.8
	Rosuvastatin	104.4 ± 3.0	120.3 ± 4.0
Blood clotting	Clopidogrel	89.7 ± 1.2	97.2 ± 3.4
	Rivaroxaban	105.8 ± 2.2	99.8 ± 2.3
	Ticlopidine	93.2 ± 7.8	79.3 ± 10.5
Type 2 diabetes	Acarbose	72.8 ± 4.6	68.2 ± 0.8
	Metformin	92.9 ± 1.3	94.8 ± 0.8
	Repaglinide	104.4 ± 1.7	109.3 ± 9.8
	Sitagliptin	94.6 ± 4.8	99.4 ± 5.5
Pain	Acetaminophen	92.6 ± 1.8	94.4 ± 1.7
	Ibuprofen	89.4 ± 2.1	89.2 ± 1.9
Hypothyroidism	Levothyroxine	95.8 ± 5.5	97.7 ± 2.6
Contraception	Drospirenone *	79.9 ± 4.8	97.8 ± 3.7
	Ethinyl estradiol	98.2 ± 10.6	90.7 ± 2.5

* Significant differences (*p* < 0.05) between GelA and GelB.

## Data Availability

The original contributions presented in this study are included in the manuscript and in the Supplementary Materials. Further inquiries can be directed to the corresponding authors.

## Supplementary Materials

The following supporting information can be downloaded at https://www.mdpi.com/article/10.3390/pharmaceutics18091173/s1, Figure S1: Calibration curves used for UV-Vis drug quantification under simulated intestinal conditions; Figure S2: Drug recovery over time, measured in Step 3 of the in vitro assay; Table S1: Compositions of stock solutions prepared for the in vitro assay; Table S2: Experimental parameters utilized for drug dissolution and UV-Vis quantification.
