# Supplementary material for: Non-Systemic Oral Cellulose-Based Superabsorbent Hydrogels for Weight Management: Potential Drug-Device Interactions Estimated by a Simplified In Vitro Assay
Source: Pharmaceutics. 2026 Sep 17;18(9):1173. doi: 10.3390/pharmaceutics18091173 (PMC13610608; doi:10.3390/pharmaceutics18091173)
Supplement: Supplementary file 1 [file pharmaceutics-18-01173-s001.zip › pharmaceutics-4487625-supplementary.pdf]

Supplementary Information

Non-Systemic Oral Cellulose-Based Superabsorbent Hydrogels for Weight Management: Potential Drug-Device Interactions Estimated by a Simplified In Vitro Assay

Eliana Panteca [1,*,†], Ivo Surano [1,†], Christian Demitri [2], Yishai Zohar [1], Marta Madaghiele [2,*] and Alessandro Sannino [2]

[1] Gelesis, 73021 Calimera, Italy; isurano@gelesis.com (I.S.); yishaizohar@gmail.com (Y.Z.)

[2] Department of Experimental Medicine, University of Salento, 73100 Lecce, Italy; christian.demitri@unisalento.it (C.D.); alessandro.sannino@unisalento.it (A.S.)

* Correspondence: epanteca@gelesis.com (E.P.); marta.madaghiele@unisalento.it (M.M.)

† These authors contributed equally to this work.

Supplementary Table S1. Compositions of stock solutions prepared for the simplified in vitro assay. Simulated Gastric Fluid (SGF) and Simulated Intestinal Fluid (SIF).

	SGF	SIF
**COMPOSITION**		
Sodium chloride (g)	2.0	-
Hydrochloric acid 1 M (mL)	84.0	-
Purified water (mL)	-	750.0
Monobasic potassium phosphate (g)	-	6.8
Sodium hydroxide 0.2 N (g)	-	117.0
Adjust pH and/or volume		
Sodium hydroxide 0.2 N to (or hydrochloric acid 0.2N)	-	pH 6.8
Purified water to (L)	1.0	1.0
**PROPERTIES**		
pH	1.1	6.8
Ionic strenght (mM)	118	72

Supplementary Table S2. Experimental parameters utilized for drug dissolution and UV-Vis quantification.
For each drug substance, pre-dissolution in organic solvents (before dissolution in diluted SGF, i.e., SGF/water 1/8 v/v) and/or the use of surfactants is indicated, if any, along with the wavelength (λ) and the dilution ratio adopted for detection and quantification, under simulated gastric and intestinal conditions. Where noted, the UV-Vis absorbance of the CMC released by a given CB-SAH (either GelA or GelB) was subtracted from the absorbance of the experimental sample (at Steps 3 and 4) to correctly estimate the drug amount. SLS = sodium lauryl sulfate; PP = potassium permanganate.

Therapeutic Indication	Drug substance	Changes to dissolution medium	Dissolution time (min)	UV-Vis detection and quantitation				
				Gastric conditions (Step 1)		Intestinal conditions (Steps 3 and 4)		Additional notes
				Dilution ratio	λ (nm)	Dilution ratio	λ (nm)	
Hypertension	Atenolol	/	30	/	275	1:1.5	230	CMC interference
	Bendroflumethiazide	Pre-dissolution (5 ml methanol, sonication 40kHz, 10 min, 37°C)	15	/	275	/	275	/
	Captopril	/	30	1:3	205	1:2	205	CMC interference
	Carvedilol	/	60	1:2	285	/	285	/
	Diltiazem	/	30	1:15	235	1:3	235	/
	Furosemide	1% w/w Tween80	120	1:5	275	1:1.5	275	CMC interference
	Hydrochlorothiazide	Pre-dissolution (5 ml methanol, sonication 40kHz, 10 min, 37°C)	30	1:20	270	See notes	270	- Dilution 1:20 at Step 3<br>- Dilution 1:10 at Step 4
	Lisinopril	/	45	1:10	205	/	210	CMC interference
Dyslipidemia	Gemfibrozil	Pre-dissolution (5 ml methanol, sonication 40kHz, 10 min, 40°C) + 1% w/w Tween80	60	1:20	275	1:4	275	/
	Lovastatin	1% w/w SLS	120	1:10	240	1:2	240	CMC interference
	Rosuvastatin	/	60	1:5	240	/	240	/
Blood clotting	Clopidogrel	0.1% w/w SLS	60	/	270	/	230	/
	Rivaroxaban	1.6% w/w SLS	60	1:2	250	/	250	CMC interference
	Ticlopidine	1% w/w SLS in SIF only	30	/	270	/	270	CMC interference

<table>
<thead>
<tr><th rowspan="3">Therapeutic Indication</th><th rowspan="3">Drug substance</th><th rowspan="3">Changes to dissolution medium</th><th rowspan="3">Dissolution time (min)</th><th colspan="5">UV-Vis detection and quantitation</th></tr>
<tr><th colspan="2">Gastric conditions (Step 1)</th><th colspan="2">Intestinal conditions (Steps 3 and 4)</th><th rowspan="2">Additional notes</th></tr>
<tr><th>Dilution ratio</th><th>λ (nm)</th><th>Dilution ratio</th><th>λ (nm)</th></tr>
</thead>
<tbody>
<tr><td rowspan="4">Type 2 diabetes</td><td>Acarbose</td><td>/</td><td>60</td><td>1:20</td><td>430</td><td>3:10</td><td>430</td><td>- Need of PP for detection, as described by Ibrahim et al., Int J Biomed Sci 3(1): 20-30, 2007
- CMC interference</td></tr>
<tr><td>Metformin</td><td>/</td><td>60</td><td>1:100</td><td>230</td><td>1:100</td><td>235</td><td>/</td></tr>
<tr><td>Repaglinide</td><td>/</td><td>30</td><td>/</td><td>240</td><td>/</td><td>240</td><td>/</td></tr>
<tr><td>Sitagliptin</td><td>/</td><td>60</td><td>/</td><td>265</td><td>/</td><td>265</td><td>/</td></tr>
<tr><td rowspan="2">Pain</td><td>Acetaminophen</td><td>/</td><td>60</td><td>1:200</td><td>245</td><td>1:40</td><td>245</td><td>/</td></tr>
<tr><td>Ibuprofen</td><td>1% w/w SLS</td><td>120</td><td>1:2</td><td>265</td><td>1:5</td><td>230</td><td>CMC interference</td></tr>
<tr><td>Hypothyroidism</td><td>Levothyroxine</td><td>1% w/w SLS</td><td>120</td><td>/</td><td>230</td><td>/</td><td>230</td><td>CMC interference</td></tr>
<tr><td rowspan="2">Contraception</td><td>Drospirenone</td><td>1% w/w SLS</td><td>Overnight</td><td>1:4</td><td>275</td><td>See notes</td><td>275</td><td rowspan="2">- Simultaneous analysis of both active principles, with CMC interference (especially at 220 nm)
- GelA: dilution 1:2 at Step 3 and no dilution at Step 4
- GelB: no dilution at both Steps 3 and 4</td></tr>
<tr><td>Ethinyl Estradiol</td><td>1% w/w SLS</td><td>Overnight</td><td>1:4</td><td>220</td><td>See notes</td><td>220</td></tr>
</tbody>
</table>

Supplementary Figure S1. Calibration curves used for UV-Vis drug quantification under simulated intestinal conditions. Unless otherwise specified, for each drug the same calibration curve was used at Steps 3 and 4 of the simplified in vitro assay, with both GelA and GelB. Further information on the UV-Vis analytical method is provided in the Supplementary Table S2.

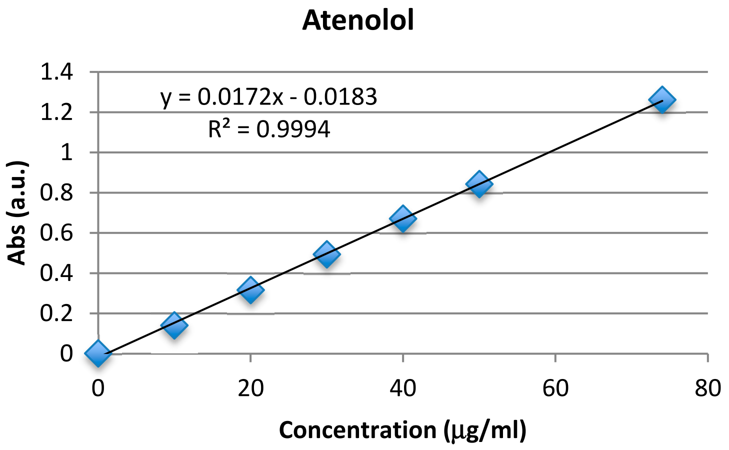

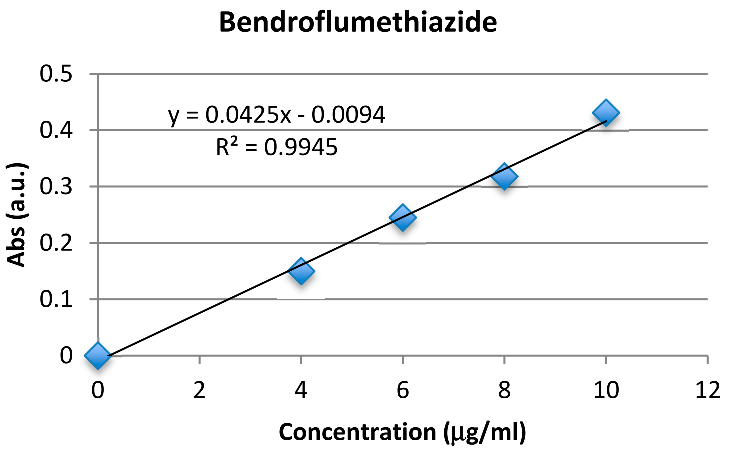

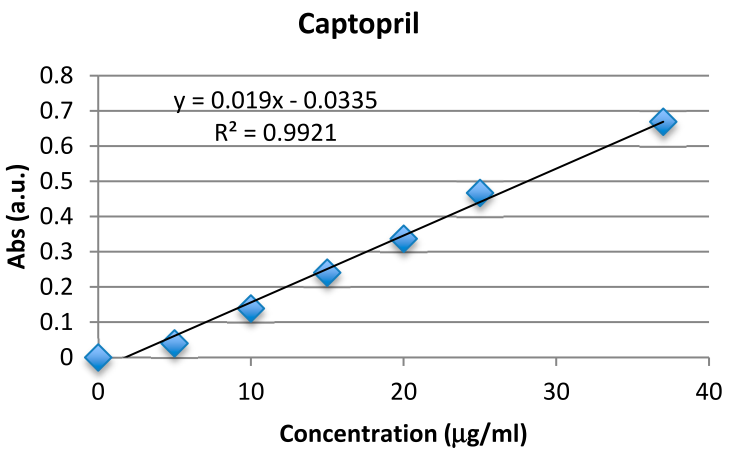

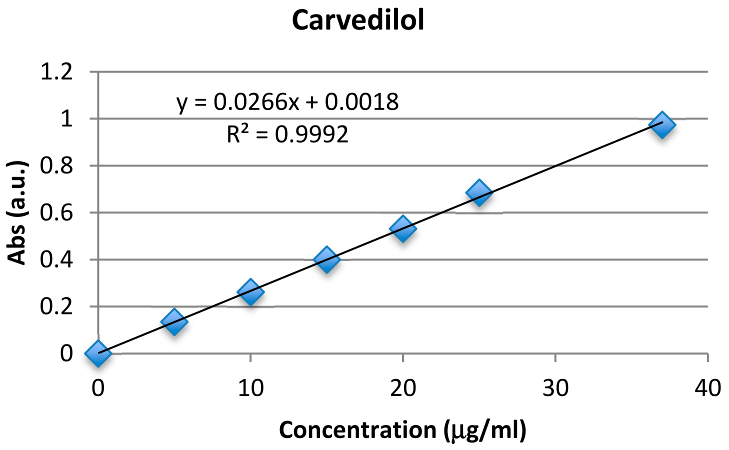
Carvedilol
y = 0.0266x + 0.0018
R² = 0.9992
Abs (a.u.)
1.2
1
0.8
0.6
0.4
0.2
0
0
10
20
30
40
Concentration (μg/ml)

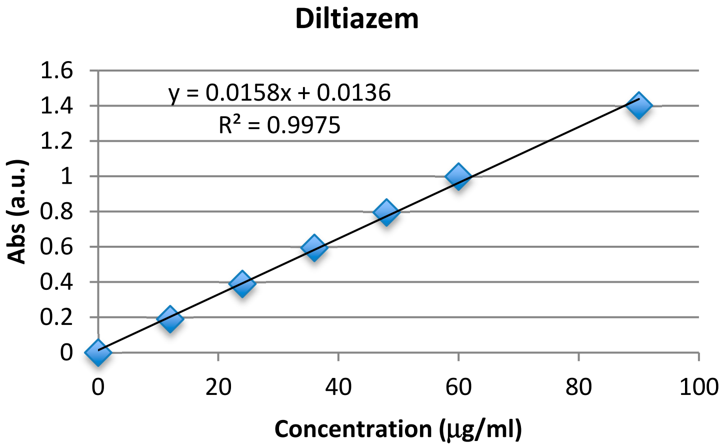
Diltiazem
y = 0.0158x + 0.0136
R² = 0.9975
Abs (a.u.)
1.6
1.4
1.2
1
0.8
0.6
0.4
0.2
0
0
20
40
60
80
100
Concentration (μg/ml)

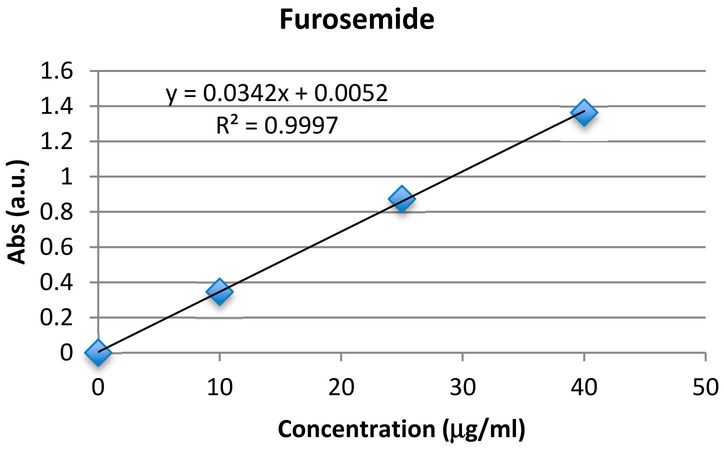
Furosemide
y = 0.0342x + 0.0052
R² = 0.9997
Abs (a.u.)
1.6
1.4
1.2
1
0.8
0.6
0.4
0.2
0
0
10
20
30
40
50
Concentration (μg/ml)

Hydrochlorothiazide Step 3

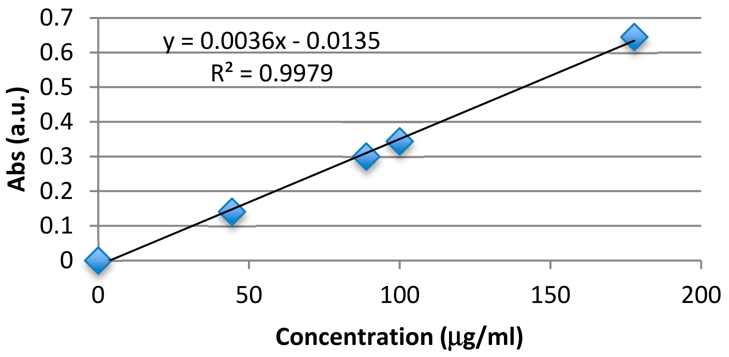

Hydrochlorothiazide Step 4

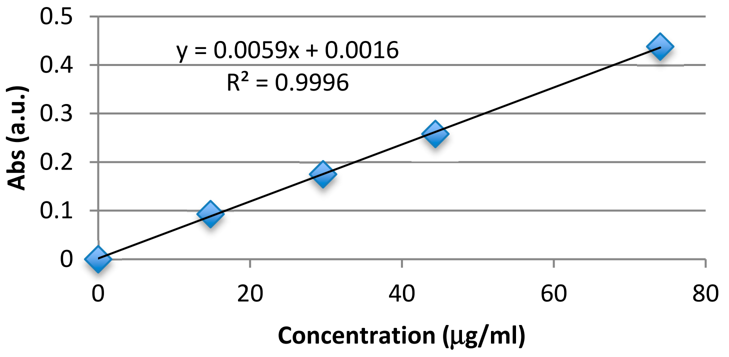

Lisinopril

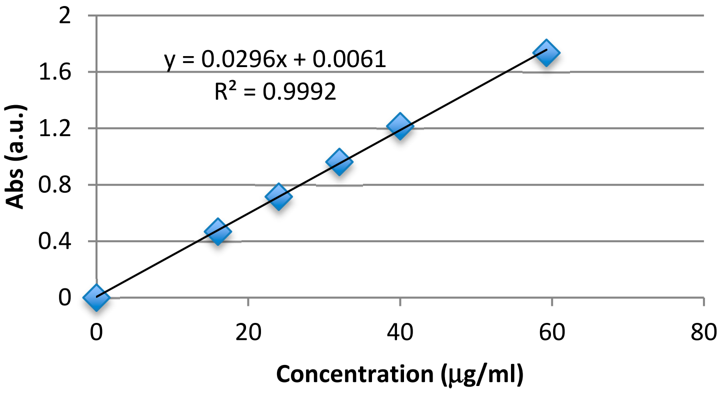

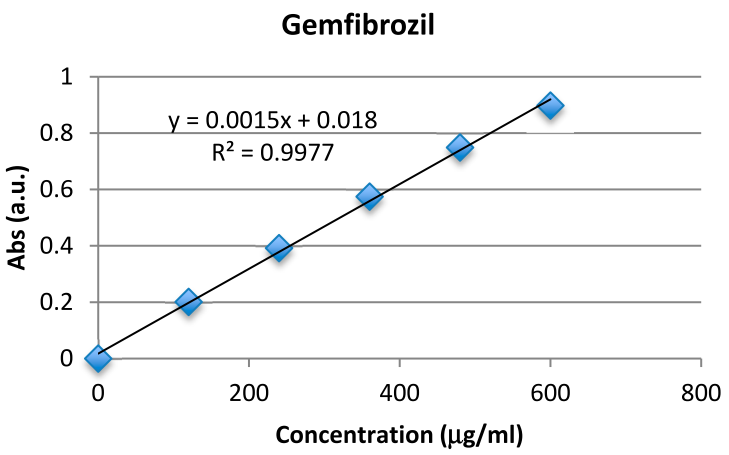
Gemfibrozil
y = 0.0015x + 0.018
R² = 0.9977
Abs (a.u.)
Concentration (µg/ml)
0
0.2
0.4
0.6
0.8
1
0
200
400
600
800

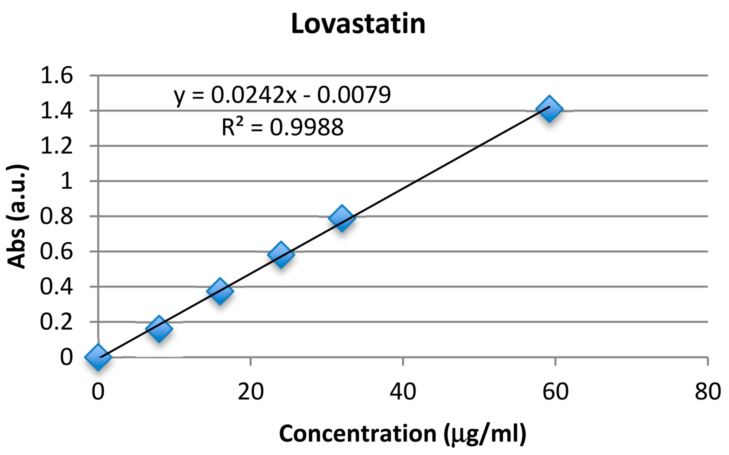
Lovastatin
y = 0.0242x - 0.0079
R² = 0.9988
Abs (a.u.)
Concentration (µg/ml)
0
0.2
0.4
0.6
0.8
1
1.2
1.4
1.6
0
20
40
60
80

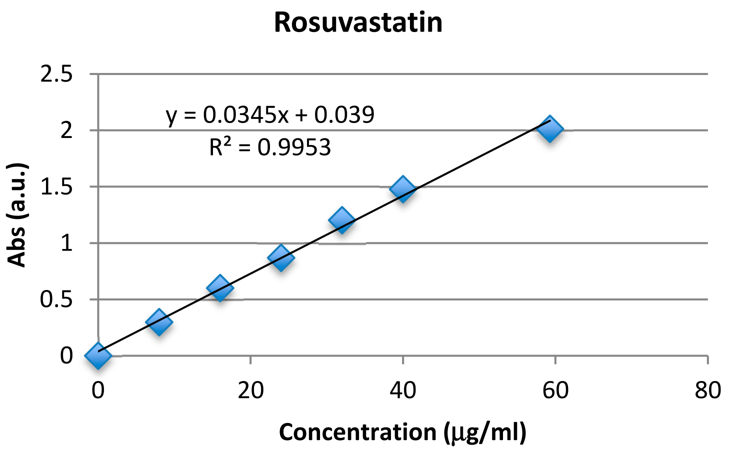
Rosuvastatin
y = 0.0345x + 0.039
R² = 0.9953
Abs (a.u.)
Concentration (µg/ml)
0
0.5
1
1.5
2
2.5
0
20
40
60
80

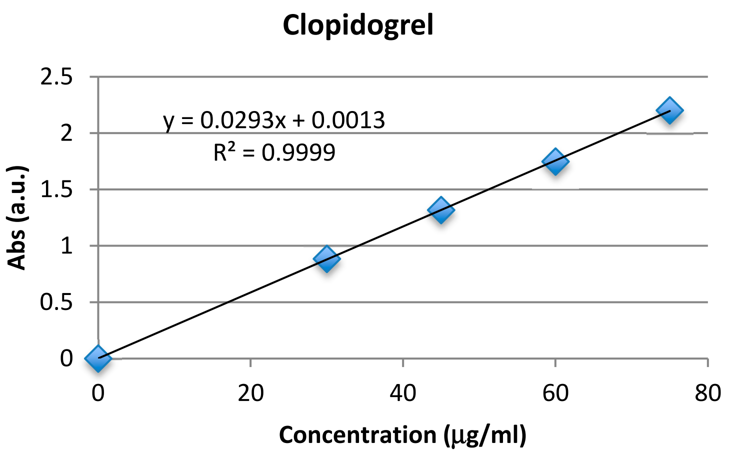
Clopidogrel
y = 0.0293x + 0.0013
R² = 0.9999
Abs (a.u.)
2.5
2
1.5
1
0.5
0
0
20
40
60
80
Concentration (µg/ml)

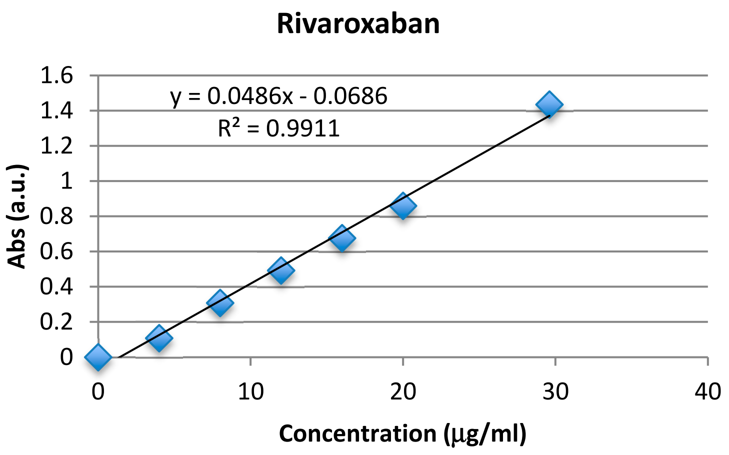
Rivaroxaban
y = 0.0486x - 0.0686
R² = 0.9911
Abs (a.u.)
1.6
1.4
1.2
1
0.8
0.6
0.4
0.2
0
0
10
20
30
40
Concentration (µg/ml)

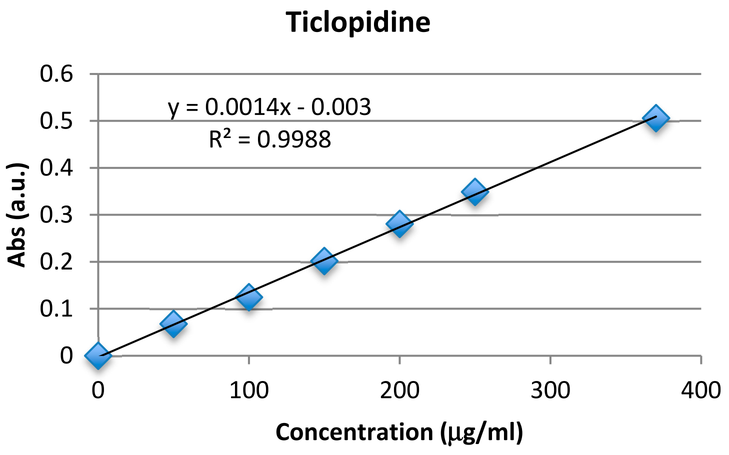
Ticlopidine
y = 0.0014x - 0.003
R² = 0.9988
Abs (a.u.)
0.6
0.5
0.4
0.3
0.2
0.1
0
0
100
200
300
400
Concentration (µg/ml)

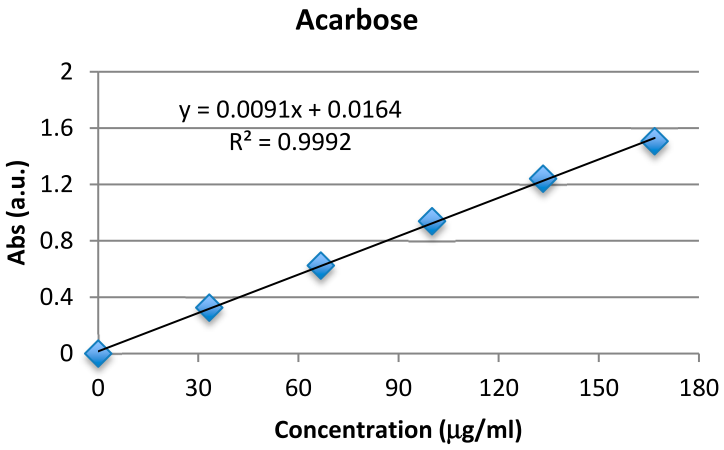
Acarbose
y = 0.0091x + 0.0164
R² = 0.9992
Abs (a.u.)
2
1.6
1.2
0.8
0.4
0
0
30
60
90
120
150
180
Concentration (µg/ml)

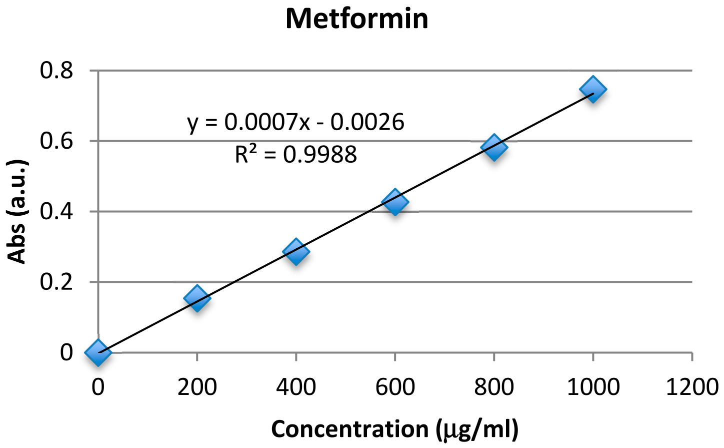
Metformin
y = 0.0007x - 0.0026
R² = 0.9988
Abs (a.u.)
0.8
0.6
0.4
0.2
0
0
200
400
600
800
1000
1200
Concentration (µg/ml)

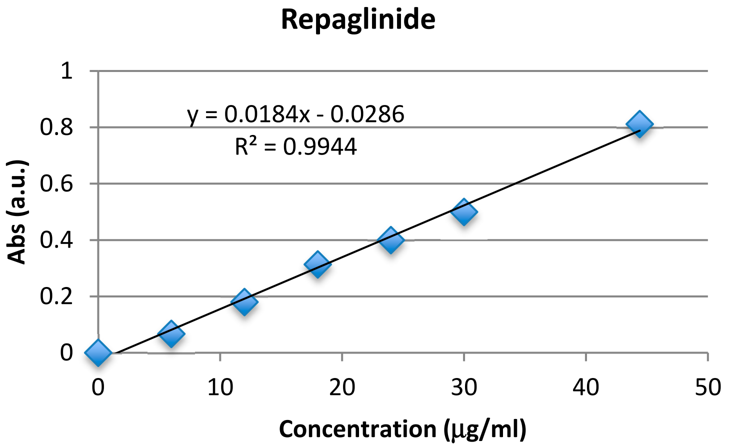
Repaglinide
y = 0.0184x - 0.0286
R² = 0.9944
Abs (a.u.)
1
0.8
0.6
0.4
0.2
0
0
10
20
30
40
50
Concentration (µg/ml)

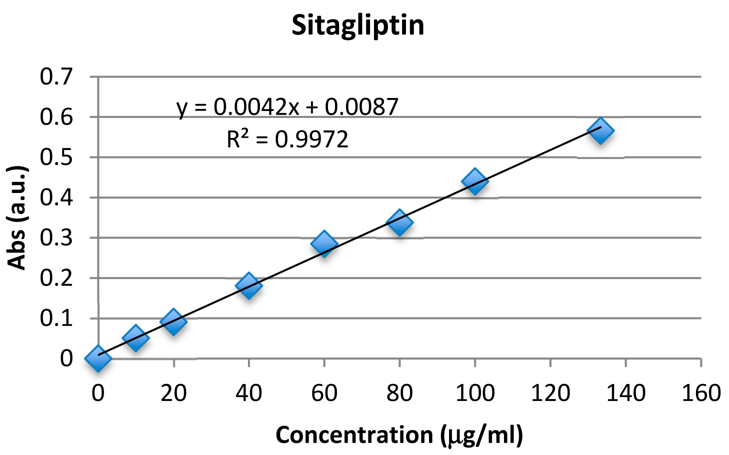
Sitagliptin
y = 0.0042x + 0.0087
R² = 0.9972
Abs (a.u.)
0.7
0.6
0.5
0.4
0.3
0.2
0.1
0
0 20 40 60 80 100 120 140 160
Concentration (µg/ml)

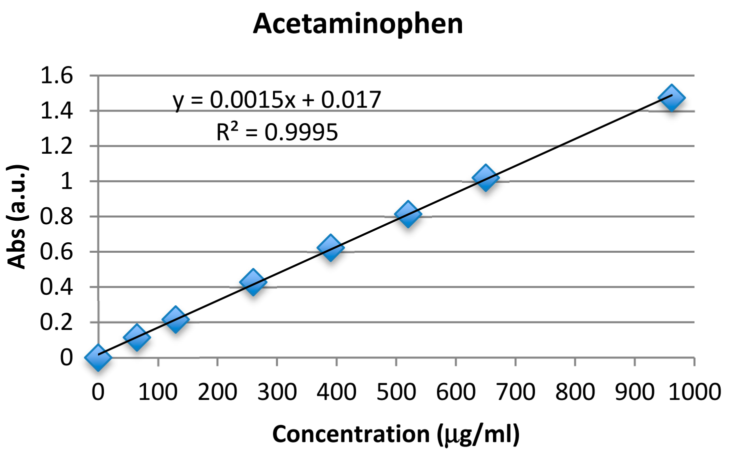
Acetaminophen
y = 0.0015x + 0.017
R² = 0.9995
Abs (a.u.)
1.6
1.4
1.2
1
0.8
0.6
0.4
0.2
0
0 100 200 300 400 500 600 700 800 900 1000
Concentration (µg/ml)

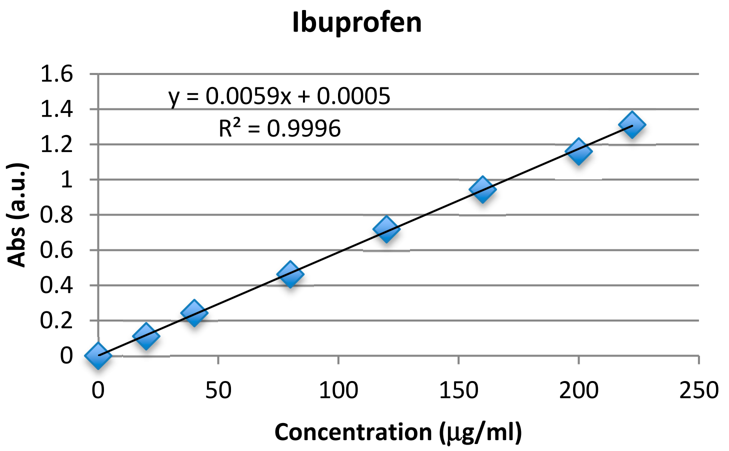
Ibuprofen
y = 0.0059x + 0.0005
R² = 0.9996
Abs (a.u.)
1.6
1.4
1.2
1
0.8
0.6
0.4
0.2
0
0 50 100 150 200 250
Concentration (µg/ml)

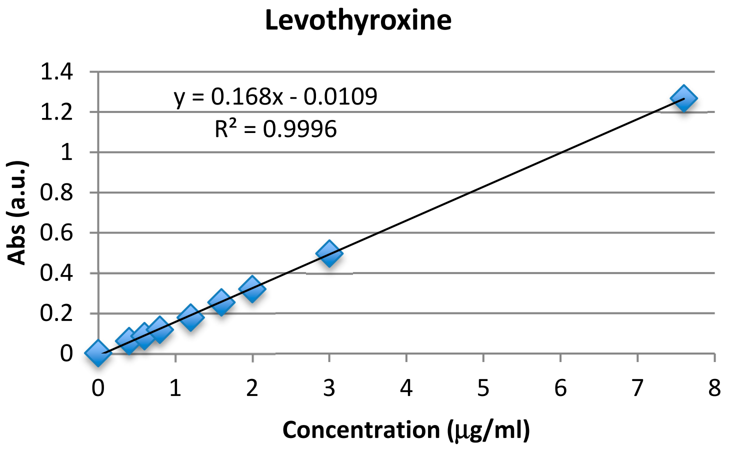
Levothyroxine
y = 0.168x - 0.0109
R² = 0.9996
Abs (a.u.)
Concentration (µg/ml)

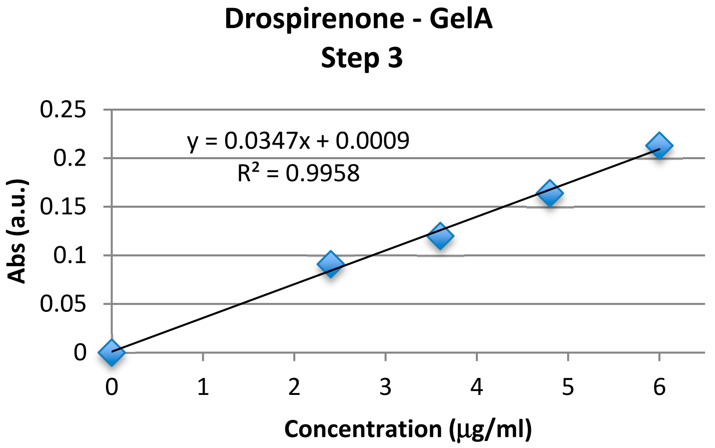
Drospirenone - GelA
Step 3
y = 0.0347x + 0.0009
R² = 0.9958
Abs (a.u.)
Concentration (µg/ml)

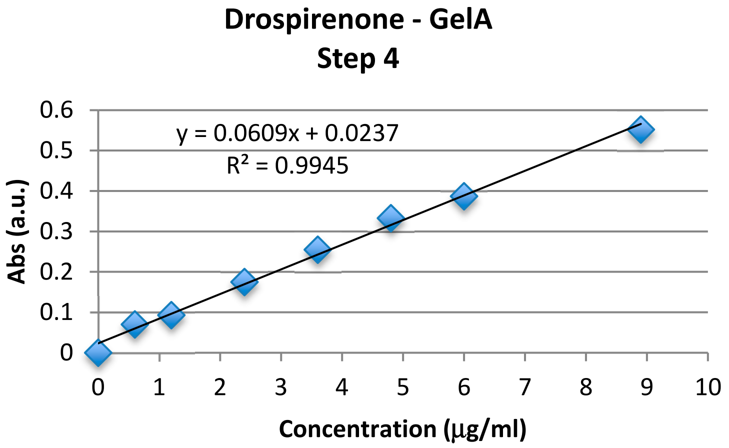
Drospirenone - GelA
Step 4
y = 0.0609x + 0.0237
R² = 0.9945
Abs (a.u.)
Concentration (µg/ml)

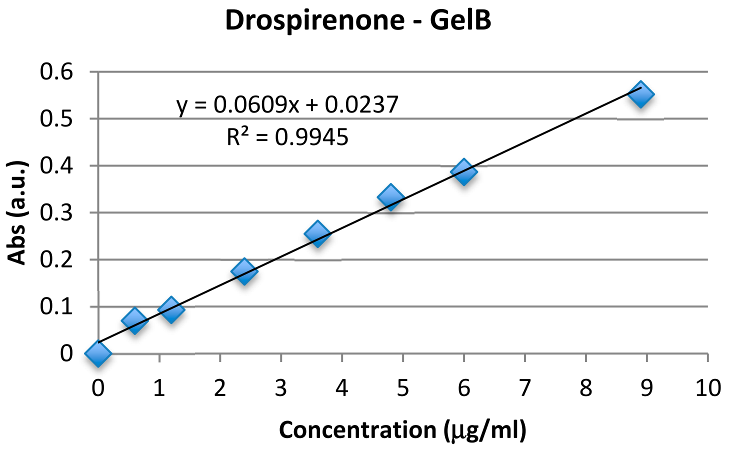

Drospirenone - GelB
y = 0.0609x + 0.0237
R² = 0.9945
Abs (a.u.)
Concentration (μg/ml)
0
0.1
0.2
0.3
0.4
0.5
0.6
0
1
2
3
4
5
6
7
8
9
10

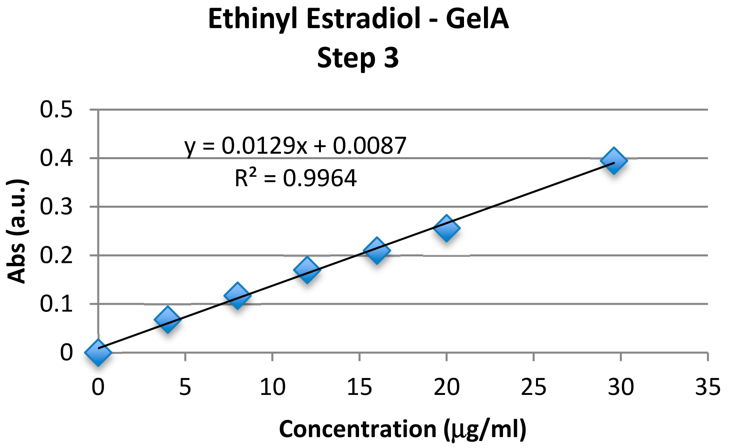

Ethinyl Estradiol - GelA
Step 3
y = 0.0129x + 0.0087
R² = 0.9964
Abs (a.u.)
Concentration (μg/ml)
0
0.1
0.2
0.3
0.4
0.5
0
5
10
15
20
25
30
35

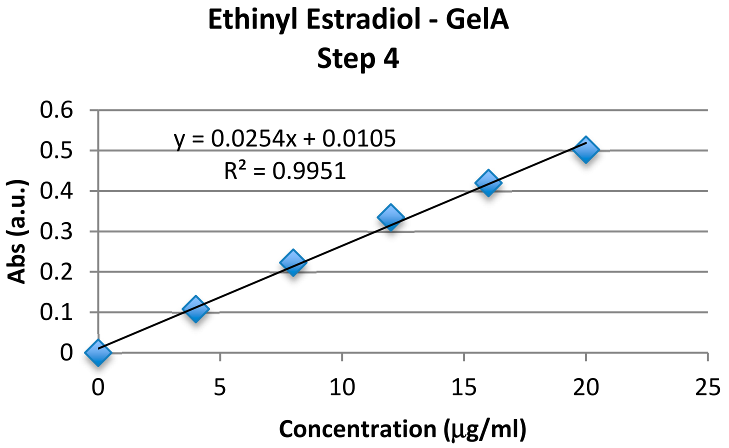

Ethinyl Estradiol - GelA
Step 4
y = 0.0254x + 0.0105
R² = 0.9951
Abs (a.u.)
Concentration (μg/ml)
0
0.1
0.2
0.3
0.4
0.5
0.6
0
5
10
15
20
25

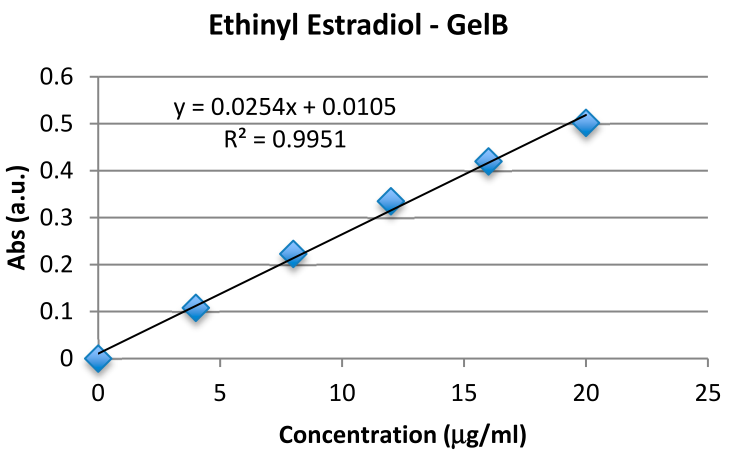
Ethinyl Estradiol - GelB
y = 0.0254x + 0.0105
R² = 0.9951
Abs (a.u.)
0.6
0.5
0.4
0.3
0.2
0.1
0
0
5
10
15
20
25
Concentration (μg/ml)

Supplementary Figure S2. Drug recovery over time measured at Step 3 of the simplified in vitro assay. For each drug, direct comparison between GelA and GelB is provided. Results are reported as mean ± SD (n=3).

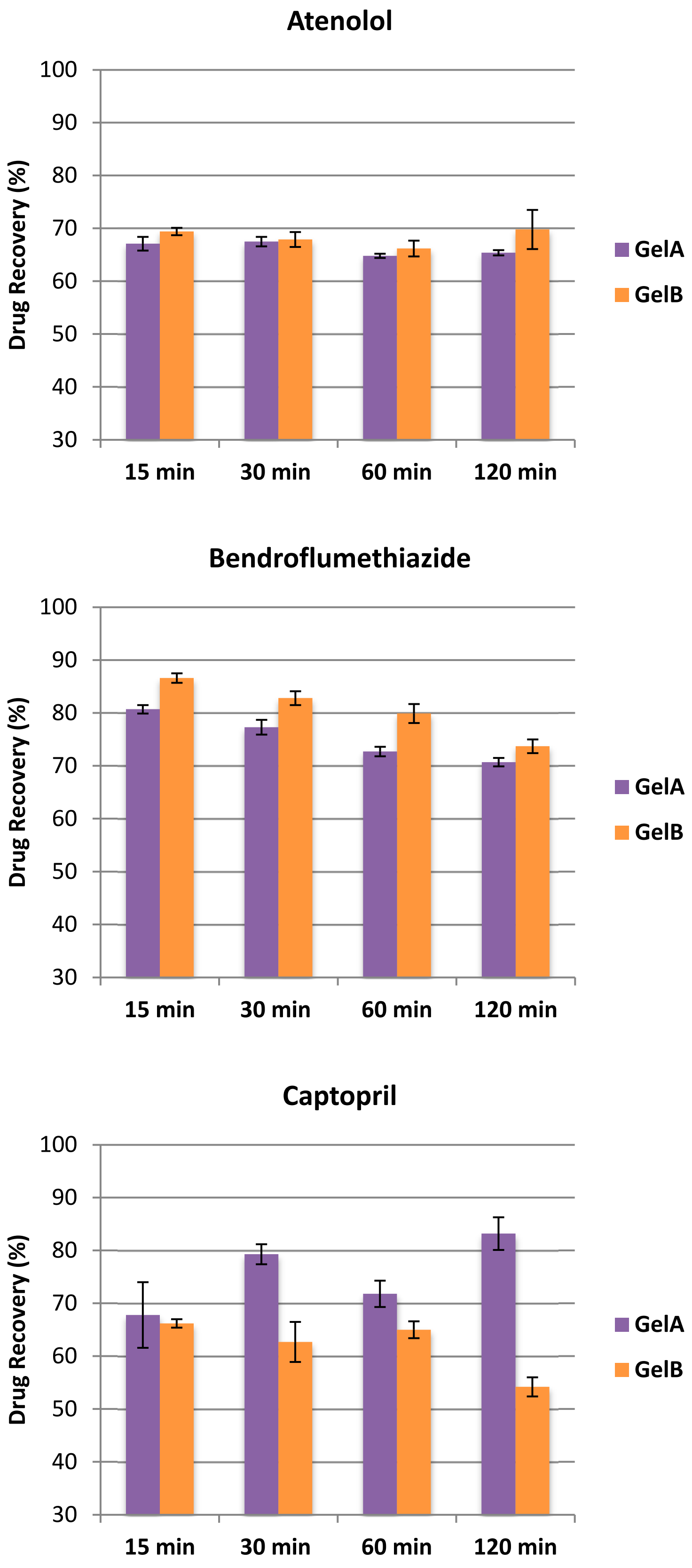

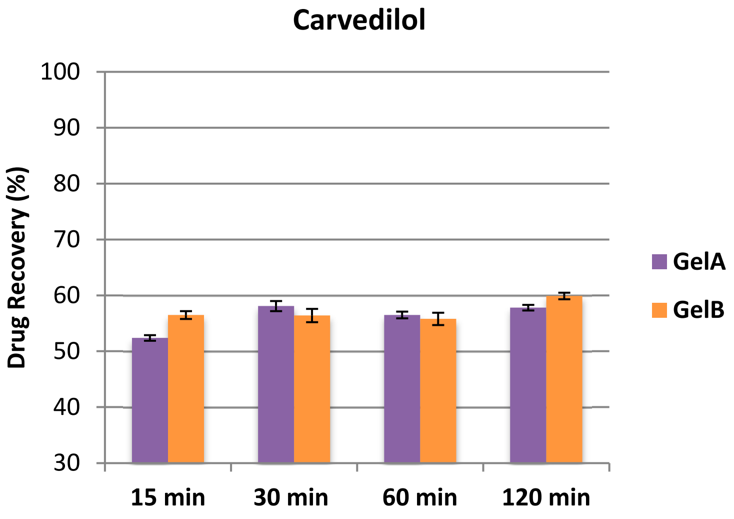
Carvedilol
Drug Recovery (%)
100
90
80
70
60
50
40
30
15 min
30 min
60 min
120 min
GelA
GelB

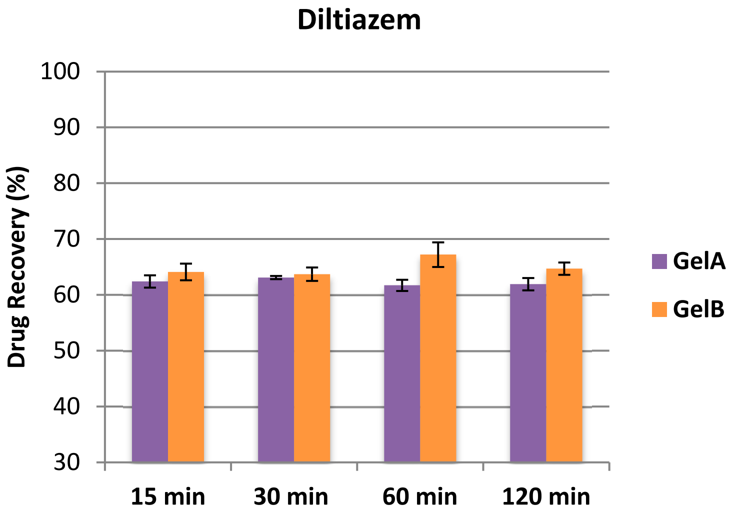
Diltiazem
Drug Recovery (%)
100
90
80
70
60
50
40
30
15 min
30 min
60 min
120 min
GelA
GelB

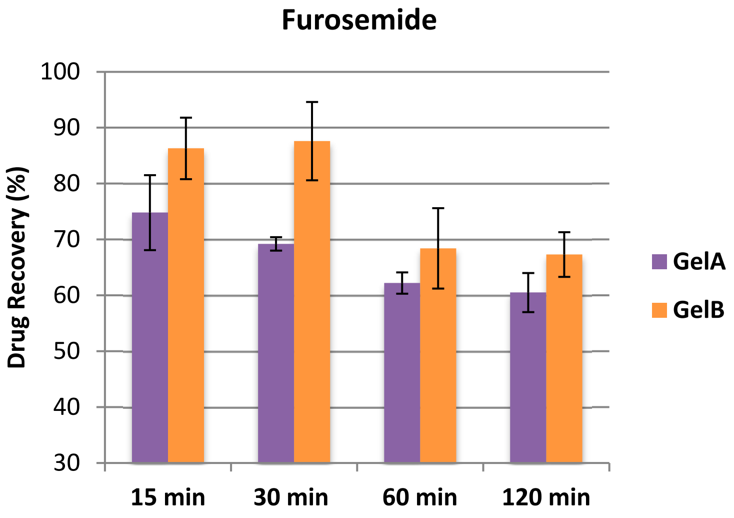
Furosemide
Drug Recovery (%)
100
90
80
70
60
50
40
30
15 min
30 min
60 min
120 min
GelA
GelB

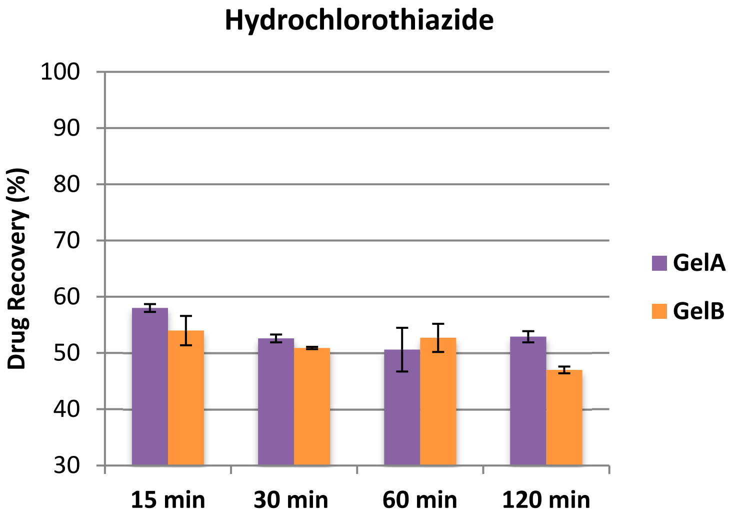
Hydrochlorothiazide
100
90
80
70
60
50
40
30
Drug Recovery (%)
15 min
30 min
60 min
120 min
GelA
GelB

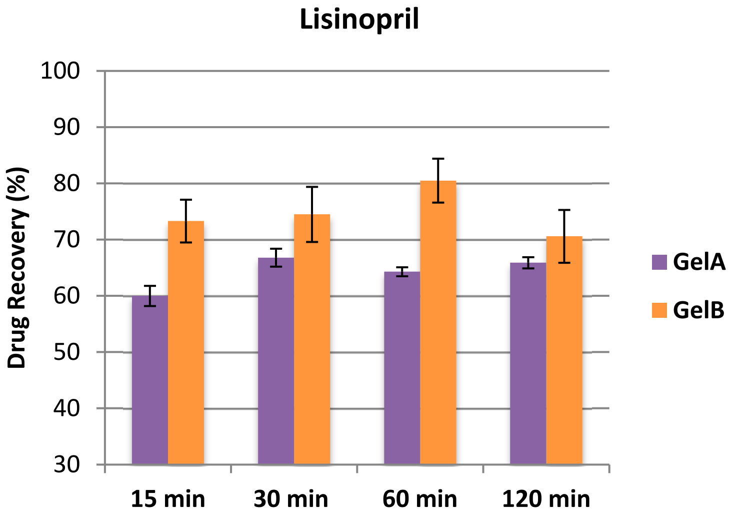
Lisinopril
100
90
80
70
60
50
40
30
Drug Recovery (%)
15 min
30 min
60 min
120 min
GelA
GelB

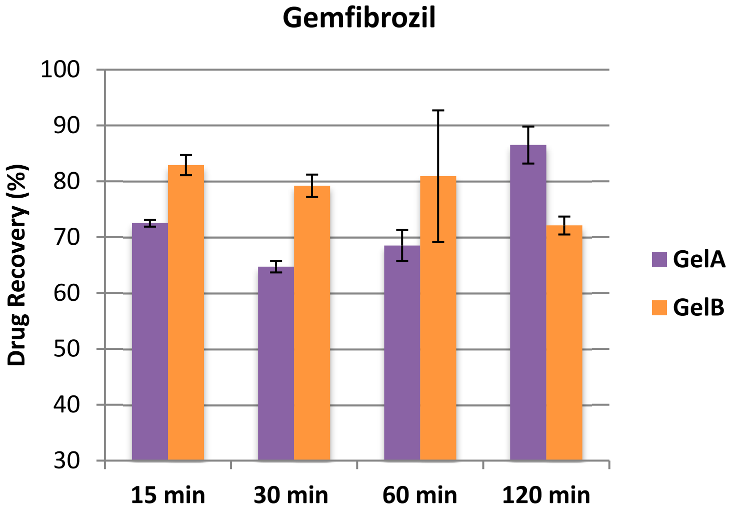
Gemfibrozil
100
90
80
70
60
50
40
30
Drug Recovery (%)
15 min
30 min
60 min
120 min
GelA
GelB

Lovastatin

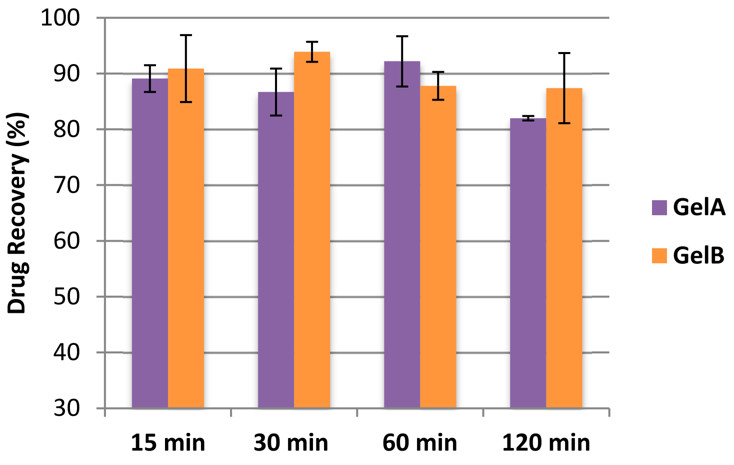

Rosuvastatin

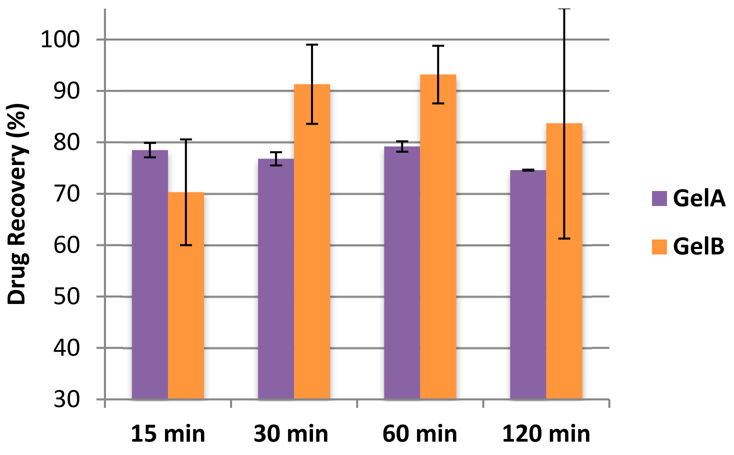

Clopidogrel

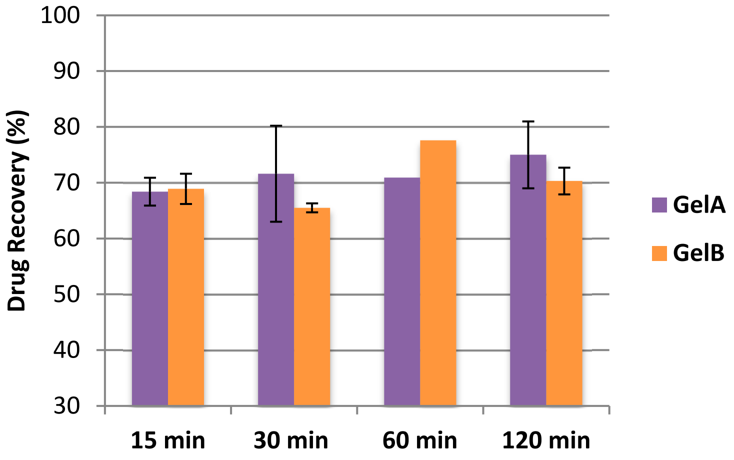

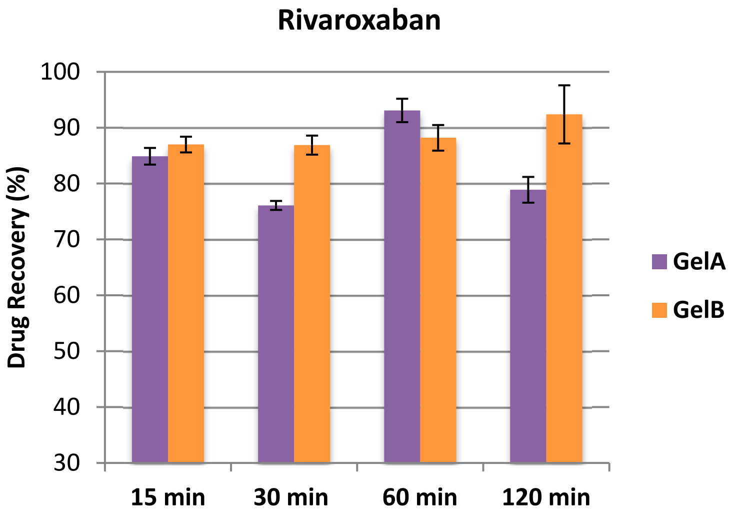
Rivaroxaban
Drug Recovery (%)
100
90
80
70
60
50
40
30
15 min
30 min
60 min
120 min
GelA
GelB

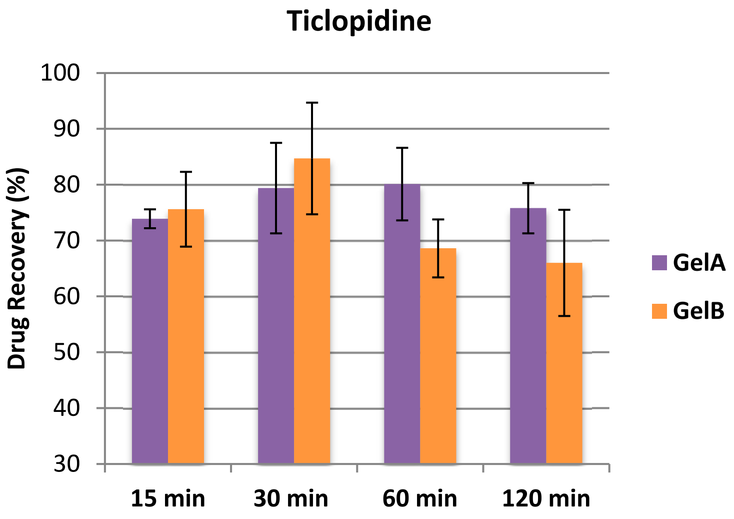
Ticlopidine
Drug Recovery (%)
100
90
80
70
60
50
40
30
15 min
30 min
60 min
120 min
GelA
GelB

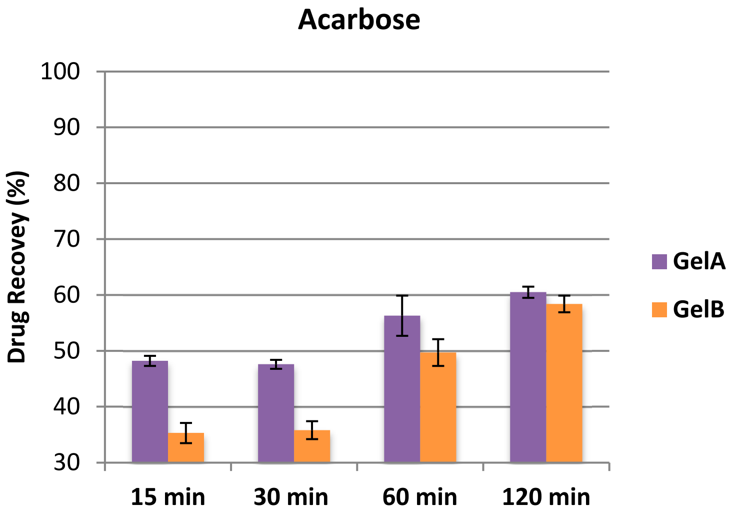
Acarbose
Drug Recovery (%)
100
90
80
70
60
50
40
30
15 min
30 min
60 min
120 min
GelA
GelB

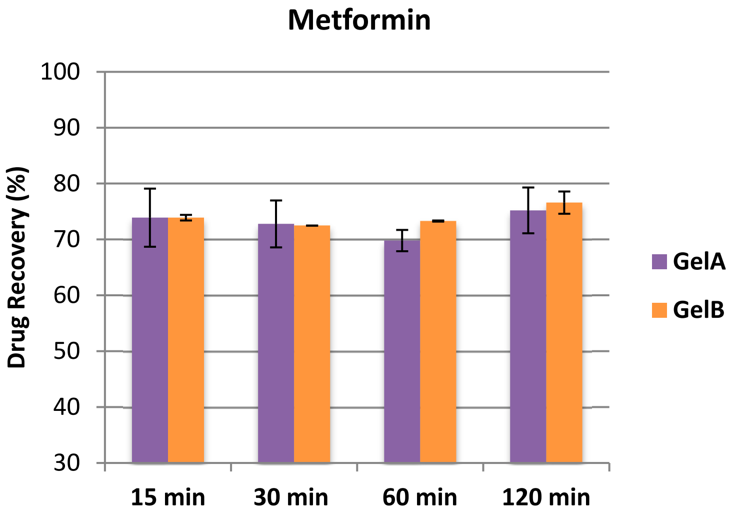
Metformin
100
90
80
70
60
50
40
30
Drug Recovery (%)
15 min
30 min
60 min
120 min
GelA
GelB

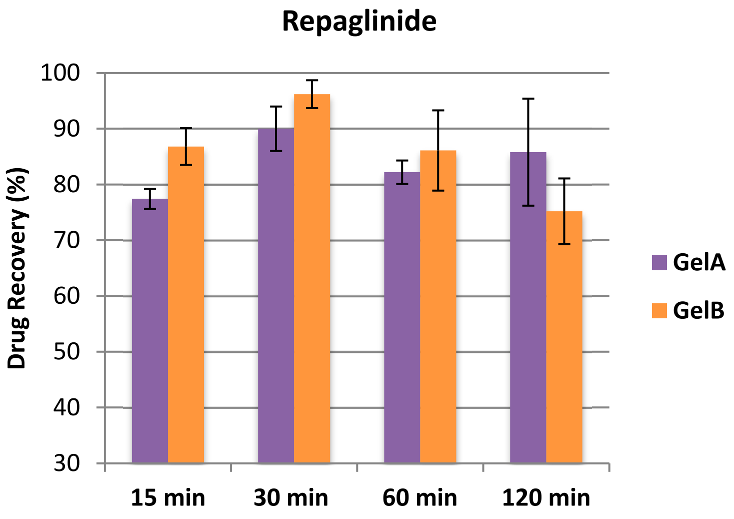
Repaglinide
100
90
80
70
60
50
40
30
Drug Recovery (%)
15 min
30 min
60 min
120 min
GelA
GelB

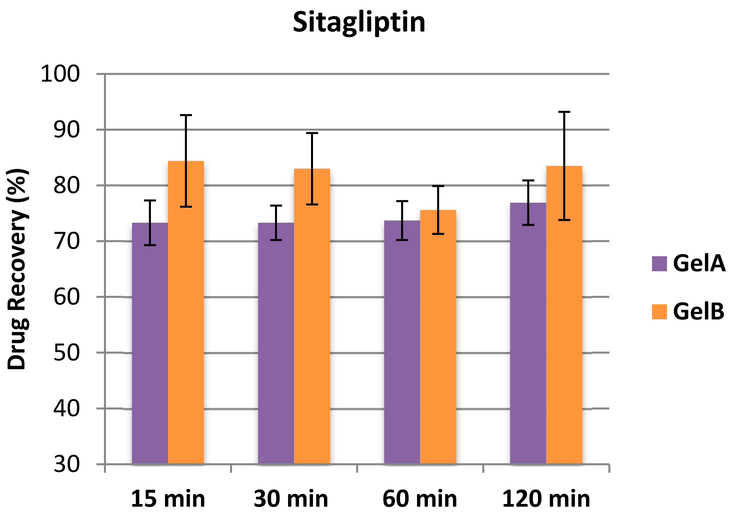
Sitagliptin
100
90
80
70
60
50
40
30
Drug Recovery (%)
15 min
30 min
60 min
120 min
GelA
GelB

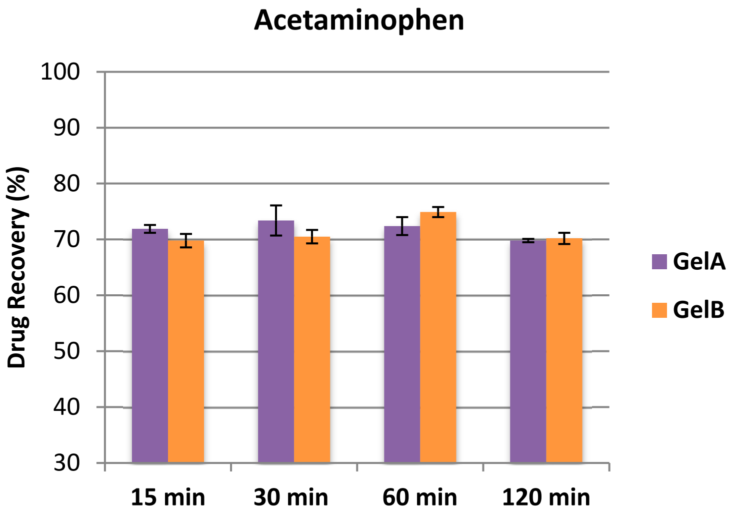
Acetaminophen
Drug Recovery (%)
100
90
80
70
60
50
40
30
15 min
30 min
60 min
120 min
GelA
GelB

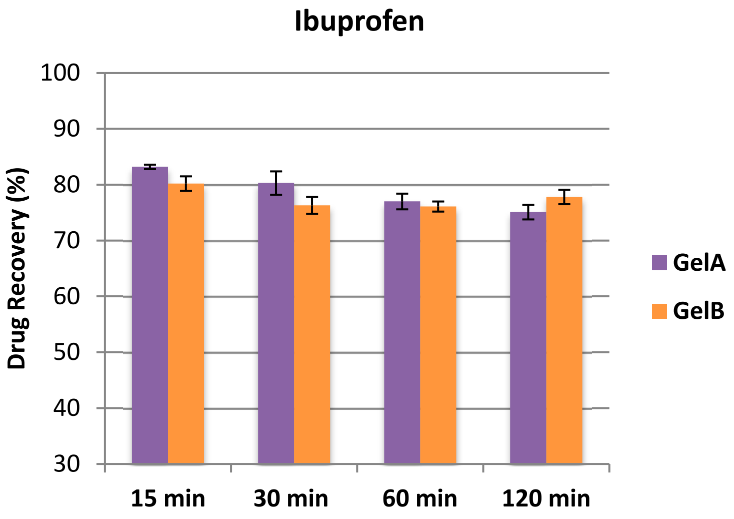
Ibuprofen
Drug Recovery (%)
100
90
80
70
60
50
40
30
15 min
30 min
60 min
120 min
GelA
GelB

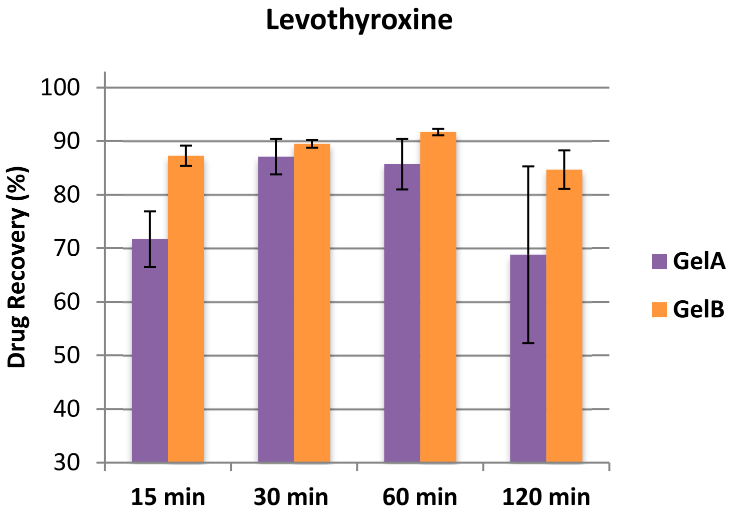
Levothyroxine
Drug Recovery (%)
100
90
80
70
60
50
40
30
15 min
30 min
60 min
120 min
GelA
GelB

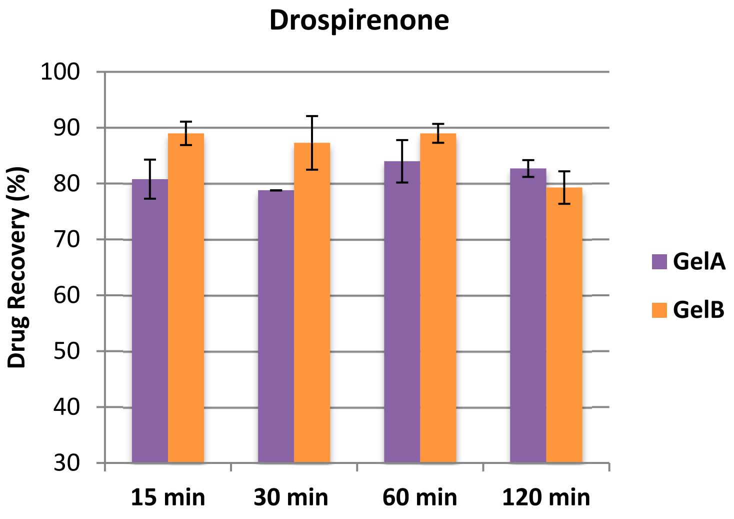
Drospirenone
Drug Recovery (%)
100
90
80
70
60
50
40
30
15 min
30 min
60 min
120 min
GelA
GelB

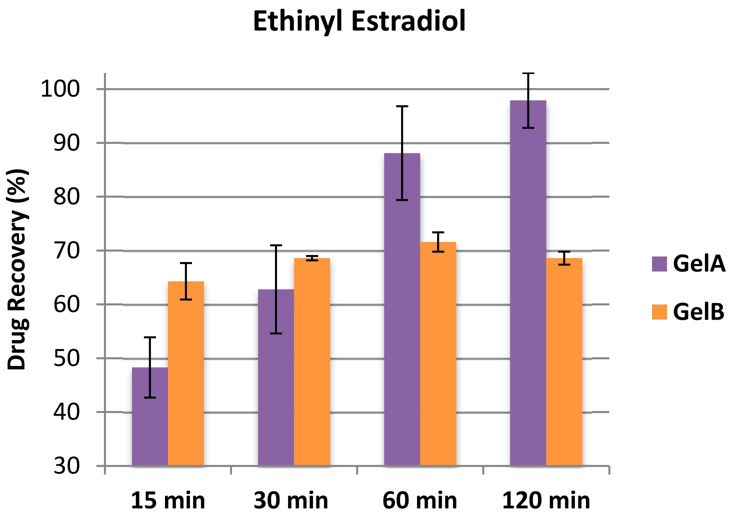
Ethinyl Estradiol
Drug Recovery (%)
100
90
80
70
60
50
40
30
15 min
30 min
60 min
120 min
GelA
GelB
